# Rational ligand modification maximizes turnover rate in a model Pd-catalyzed C-H arylation

**DOI:** 10.1016/j.isci.2022.105790

**Published:** 2022-12-10

**Authors:** Igor Beckers, Dirk De Vos

**Affiliations:** 1 KULeuven, Department of Microbial and Molecular Systems, cMACS, Celestijnenlaan 200F, Leuven 3001, Belgium

**Keywords:** Catalysis, Chemistry, Organic chemistry

## Abstract

The direct cross-coupling of (hetero)aromatics without prior functionalization is a promising reaction for the chemical and pharmaceutical industries, enabling the conversion of inexpensive feedstocks in a highly step-efficient manner. However, many C-H arylations rely on high loadings of a Pd catalyst that preclude their use in low-cost applications. In this work, we have maximized the turnover rate of a Pd-catalyzed C-H arylation reaction through rational tuning of the ligands. Starting from a computational study of the catalytic mechanism, a kinetic model was derived that accurately explains the experimental time profiles. Quantitative structure-activity relationships between the substituents on the ligands and the resulting catalytic activity for various C-H arylations were obtained. This study demonstrates that, depending on the coupling partner, the C-H activation is not the sole rate-limiting step, and that the ligands can be adapted accordingly to further accelerate catalytic turnover.

## Highlights

• Catalytic C-H arylation reaction studied via kinetic and computational modeling
• C-H activation and oxidative addition identified as rate-determining steps
• Rational modification of the ligand maximizes catalytic turnover

## Graphical abstract

**Figure:**
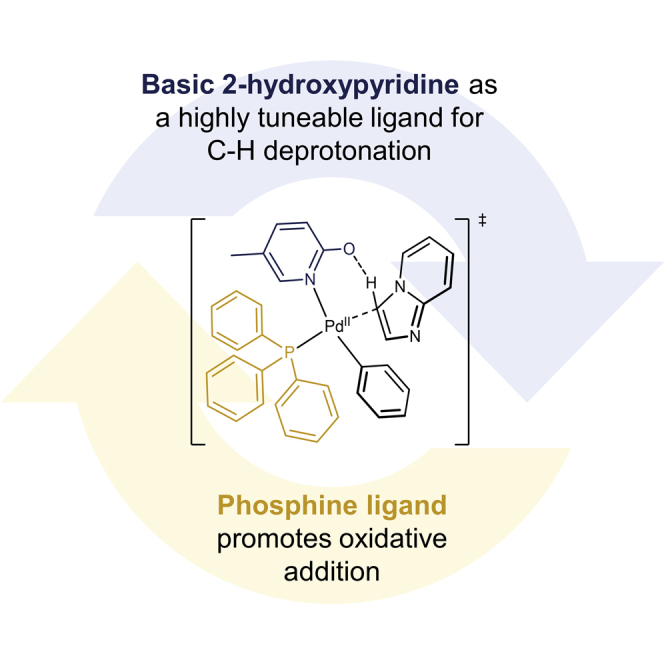

## Introduction

Pd-catalyzed cross-couplings have become essential for the industrial production of bi(hetero)aryl C-C bonds in many pharmaceuticals.^1^^,^^2^^,^^3^^,^^4^^,^^5^ Especially in drug discovery, the Suzuki and related cross-coupling reactions have been highly valuable in the preparation of numerous of bi(hetero)aryl scaffolds as potential drug candidates.^6^^,^^7^ The prevalence of the Suzuki and related reactions in preclinical phases has now translated into a large share of such bi(hetero)aryl scaffolds among marketed pharmaceuticals.^8^ While versatility (*i.e.* a large reaction scope) is a main asset on the lab scale in the pursuit of drug candidates, the focus shifts toward efficiency toward a definite synthetic target in industrial manufacture. With the most cost-efficient and sustainable route as the main goal, the catalytic activity becomes increasingly important to minimize the use of expensive Pd on scale.^9^

The direct cross-coupling via C-H activation has emerged as a promising reaction in terms of step-efficiency, omitting the need for preactivated (hetero)aryl halides and -metals.^10^^,^^11^^,^^12^ Synthetic methodologies have previously been developed that enable highly (regio)selective arylation of pharmaceutically important heteroaromatic C-H bonds with aryl halide reactants.^13^^,^^14^^,^^15^^,^^16^^,^^17^^,^^18^^,^^19^^,^^20^^,^^21^ However, many literature protocols require excessive Pd loadings that are prohibitive in larger scale applications. So far, only few direct C-H arylations have been discovered that feature very high catalytic turnover.^22^ While the loadings of Pd could be decreased to 0.1 mol %, typically under phosphine-free conditions, its catalytic activity is strongly dependent on the used (hetero)aryl halide coupling partner.^23^^,^^24^^,^^25^^,^^26^^,^^27^^,^^28^ To maintain the highest turnover numbers, these methods either rely on the use of more expensive aryl iodides or on electronically activating substituents on the aryl bromide reactant. Mechanistically, this hints on a rate-limiting oxidative addition step in the underlying catalytic cycle. With the ultimate goal of achieving the highest catalytic activity, the intimate relationship between the coupling reactants and the catalytic turnover calls for a rational framework to identify the catalytic steps that determine the overall turnover rate and to understand how these steps can be accelerated by the ligands.^29^

In this work, we report on an in-depth mechanistic investigation of the cross-coupling of *N*-heteroaromatic imidazo[1,2-*a*]pyridine with aryl bromides as a model C-H arylation reaction. A computational study of the catalytic cycle was first performed via density functional theory (DFT) calculations, highlighting both the oxidative addition and C-H activation as potential rate-determining steps. These findings were in strong agreement with kinetic experiments. A kinetic model was derived that accurately explains in the observed catalytic activity for a range of ligands and aryl bromide reactants. The Pd catalyst, bearing both a phosphine and 2-pyridone ligand, emerged as highly active and tunable catalytic system as revealed by a quantitative structure-activity relationship study (*i.e.* Hammett analysis). On the one hand, increasing the basicity of the 2-pyridone ligand strongly accelerates the C-H activation step for this reaction. On the other hand, the electron-donating phosphine ligand is beneficial for the oxidative addition. By choosing the ligand in accordance to the aryl bromide coupling partner, the high catalytic activity is retained for more challenging aryl bromides bearing deactivating substituents. These findings exemplify that the turnover rate of the Pd catalyst for C-H arylation can be maximized rationally via the modeling of the catalytic cycle and the consequent modification of the ligands.

## Results and discussion

### Computational model of the catalytic cycle

**Figure 1:**
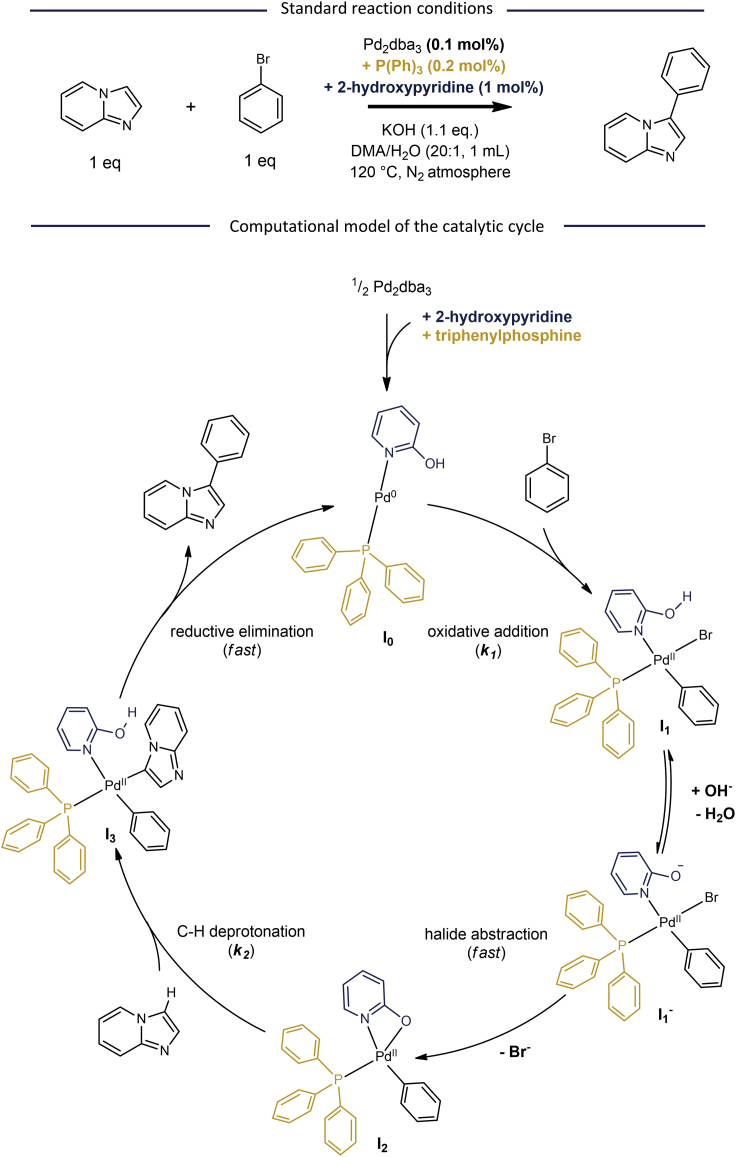
Computational model of the catalytic cycle (Top) Standard reaction conditions for the C-H arylation of imidazo[1,2-*a*]pyridine with bromobenzene investigated in this study. (Bottom) Computational model of the catalytic cycle obtained via DFT calculations. Optimization of intermediate complexes and transition states was performed B3LYP functional including dispersion corrections and solvent effects, according to a method proposed in a benchmark study by Grimme et al.^29^

To study the turnover rates for a direct C-H arylation, we focused on the regiospecific coupling of imidazo[1,2-*a*]pyridine with aryl bromides as a model reaction (Figure 1A). The highly active Pd complex bearing both a monodentate phosphine and 2-hydroxypyridine ligand served as a starting point for our computational study of the underlying catalytic mechanism. DFT calculations were performed according to a method described in a highly relevant benchmark study by Grimme et al.,^29^ which provided accurate geometrical structures of the intermediates and transition states, as well as their corresponding single-point energies (Figure 1B). After dissolution of Pd_2_dba_3_ in the reaction mixture, the Pd precursor is converted upon coordination of a triphenylphosphine and a 2-hydroxypyridine ligand to generate the active catalytic species **I**_**0**_. Similar to the traditional Suzuki cross-coupling reactions, an oxidative addition of the aryl bromide to the Pd^0^ complex (**TS**_**1**_) takes place. The computations show an active role of the 2-hydroxypyridine ligand in the halide abstraction: after its deprotonation the ligand enables a fast, intramolecular exchange of the bromide anion of complex **I**_**1**_^**−**^ via transition state **TS**_**2**_. The resulting bidentate N,O-ligand in complex **I**_**2**_ then mediates the C-H activation via a concerted metalation-deprotonation (CMD) transition state (**TS**_**3**_). A final reductive elimination (**TS**_**4**_) step generates the C-C coupled product and regenerates **I**_**0**_ from **I**_**3**_, which closes the catalytic cycle.

**Figure 2:**
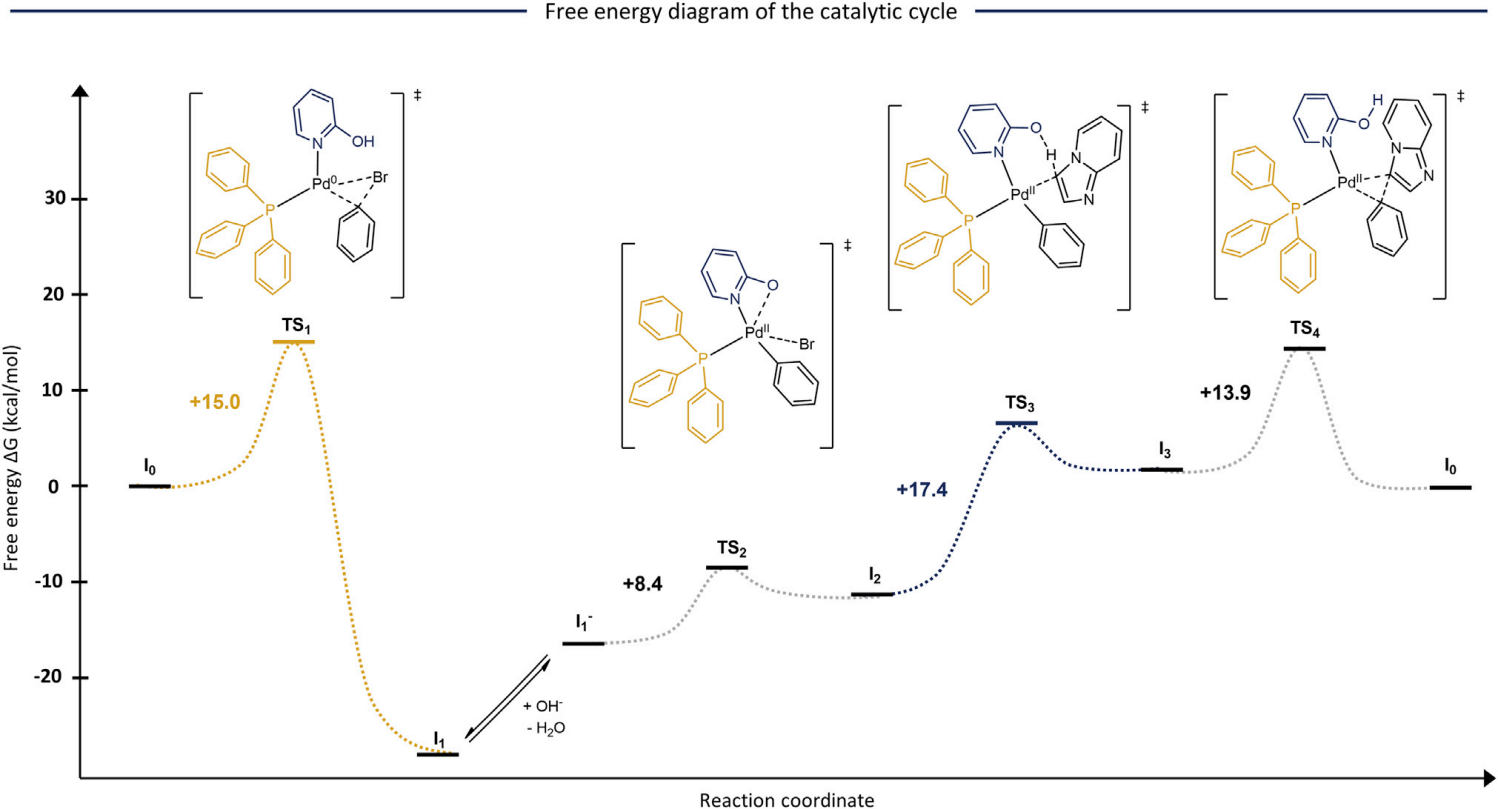
Free energy diagram of the catalytic cycle Single-point free energies (PWPB95-D3/Def2QZVPP) of the intermediates and transition states that are involved in the catalytic cycle given in kcal/mol.

Next, the corresponding single-point energies of the intermediates and transition states were calculated on the PWPB95-D3/Def2QZVPP level of theory as proposed by a relevant benchmark study.^29^ The resulting free energy diagram shows that the two highest activation energies are associated with the oxidative addition and C-H activation steps of 15.0 and 17.4 kcal/mol, respectively (Figure 2). The reductive elimination has a lower energy barrier of 13.9 kcal/mol. The model predicts that the intramolecular halide abstraction is very fast, with a low activation energy of 8.4 kcal/mol. These results indicate that the rate of the C-H activation step contributes to the overall reaction rate. Moreover, the oxidative addition also represents a considerable hurdle for fast catalytic turnover. Depending on the reactivity of the aryl bromide reactant, this step could also be rate determining. Indeed, it was previously reported in several C-H arylations that the high catalytic turnover strongly depends on the reactivity of the aryl halide coupling partner.^23^^,^^24^^,^^25^^,^^26^

### Kinetic study of the C-H arylation reaction

**Figure 3:**
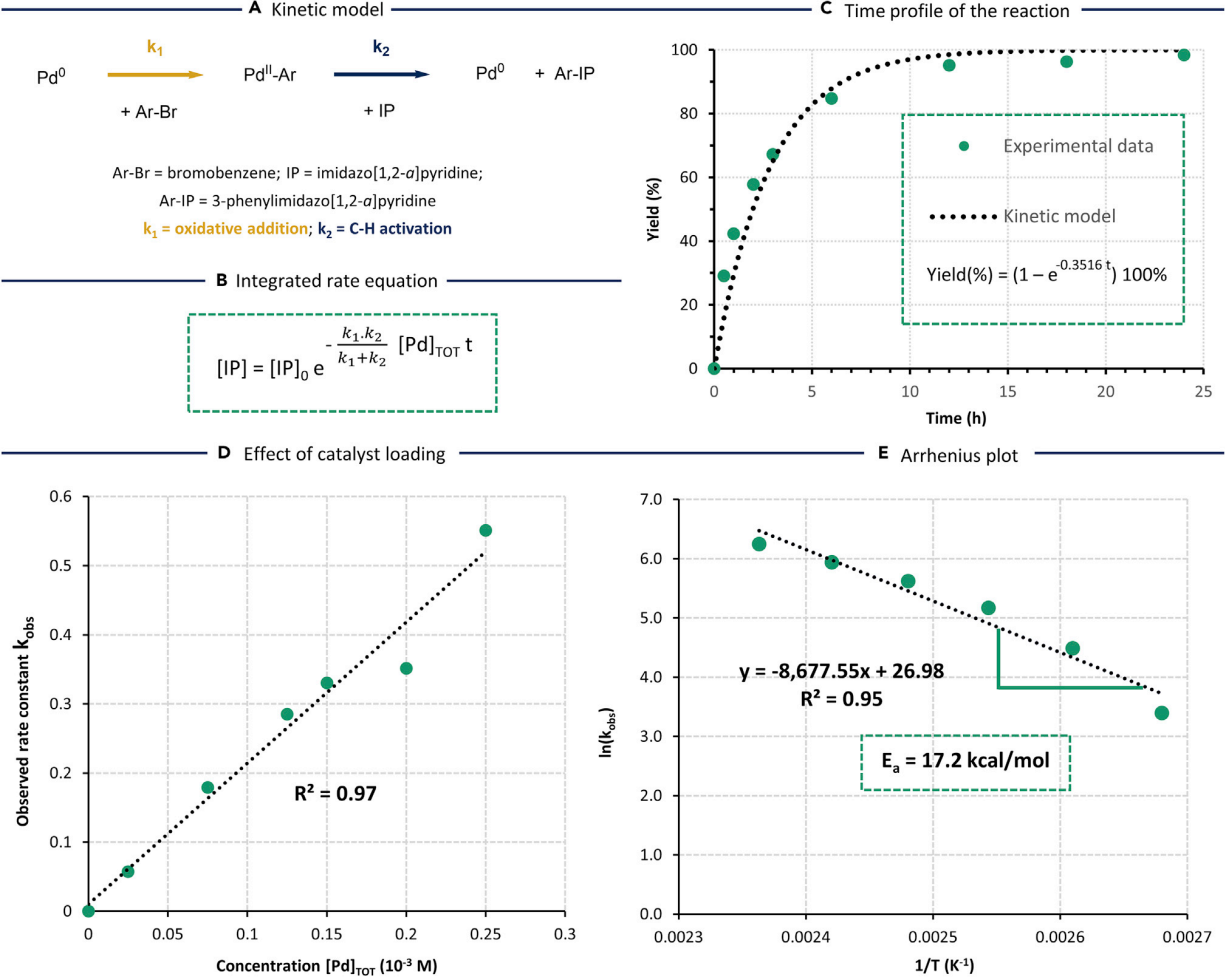
Kinetic study of the C-H arylation reaction (A) Kinetic model based on the oxidative addition and C-H activation as potential rate-determining steps. (B) The integrated rate equation derived from the kinetic model (see SI for derivation). (C) Experimentally obtained time profile of the reaction, giving the GC yield of 3-phenylimidazo[1,2-*a*]pyridine product (%) in function of time (h) and a comparison with the kinetic model. (D) Observed rate constants upon minimization of Pd catalyst concentrations. (E) Arrhenius plot of the reaction, featuring the natural logarithm of the observed rate constant in function of the inverse temperature, shows an overall activation energy of 17.2 kcal/mol.

Based on our findings in the computational study, a kinetic model was derived that accounts for both the oxidative addition and C-H activation as potentially rate-determining steps (Figure 3A). Derivation of the corresponding rate equation (see SI for derivation) reveals a first-order kinetics with an effective kinetic constant (k) that is dependent on the individual rates of oxidative addition (k_1_) and C-H activation (k_2_) (Figure S1 and Table S1). The equation shows that the overall rate is indeed strongly influenced by either the C-H activation or oxidative addition, depending on the reactivity of the aryl bromide reactant (*i.e.* k_1_) (Figure 3B). The overall rate is maximized when both steps are equally fast. The kinetic model fits the experimentally obtained time profile (Figure 3C). Next, the catalyst loading was varied from 0 to 0.25 mol % under the standard reaction conditions (Figure S6). These experiments confirmed that the reaction is first order in Pd catalyst (Figure 3D). The absence of higher order kinetics is beneficial to obtain high catalytic turnover at minimal Pd loadings below 0.2 mol %. The time profile of the reaction was also determined for various temperatures from 100°C to 150°C, showing an exponential increase in the overall reaction rate (Figure S7). The logarithm of the resulting rate constants for various temperatures is given in an Arrhenius plot (Figure 3E). Based on the linear fit of the experimental data, an overall activation energy of 17.2 kcal/mol was found, which is consistent with the highest activation barrier found in the computational model.

The rate-determining role of the C-H activation step in the overall catalytic cycle was also apparent upon comparison of the conversion rates of the imidazo[1,2-*a*]pyridine reactant with those of its deuterium-labeled analog (Figures S4–S7). A large difference in observed kinetic constants was found, showing a primary kinetic isotope effect (KIE) of 7.6. This large KIE value, which exceeds those that are usually found in the context of the CMD mechanisms with more electrophilic Pd-carboxylate catalysts,^30^ highlights the importance of the C-H deprotonation mediated by the basic 2-hydroxypyridine ligand in the corresponding transition state (**TS**_**3**_). The metalation-deprotonation mechanisms previously reported in literature are proposed to form a continuum between electrophilic metalation by the Pd center and deprotonation by the ligand.^31^^,^^32^^,^^33^ The current catalyst, bearing both an electron-donating phosphine ligand and highly basic 2-hydroxypyridine, pushes further toward the deprotonation of heteroaromatic C-H bonds.

### Structure-activity relationships between ligand and catalytic activity

**Figure 4:**
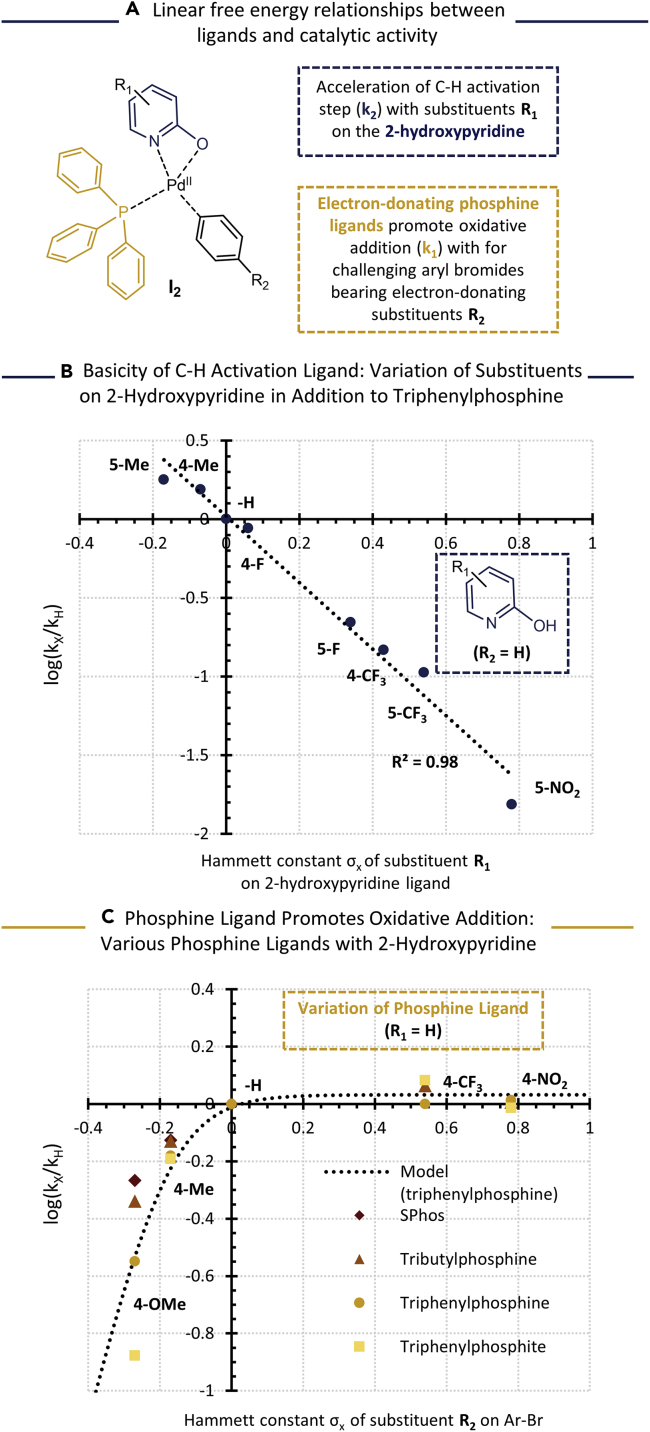
Structure-activity relationship between ligand and catalytic activity (A) General overview of the ligand modifications investigated in this study, with R_1_ the substituents on the 2-hydroxypyridine ligand (in *meta*- or *para-*position relative to the 2-hydroxy substituent), and R_2_ on the aryl bromide reactant (on *para*-position). (B) Linear free-energy relationship observed between the natural logarithm of the relative reaction rates (Figure S12) and the Hammett constant of various substituents R_1_ on the 2-hydroxypyridine ligand. Reaction conditions: Pd_2_dba_3_ (0.000125 mmol), PPh_3_ (0.00025 mmol), substituted 2-hydroxypyridine (0.00125 mmol), imidazo[1,2-*a*]pyridine (0.1 mmol), bromobenzene (0.1 mmol), KOH (0.11 mmol) in aqueous DMA (1 mL, 5 vol % H_2_O). (C) Linear free-energy relationship observed between the natural logarithm of the relative reaction rates (Figure S13 and 14) and the Hammett constant of various substituents R_2_ on the aryl bromide reactant for various phosphine ligands. Reaction conditions: Pd_2_dba_3_ (0.000125 mmol), phosphine ligand (0.00025 mmol), 2-hydroxypyridine (0.00125 mmol), imidazo[1,2-a]pyridine (0.1 mmol), 4-substituted aryl bromide reactant (0.1 mmol), KOH (0.11 mmol) in aqueous DMA (1 mL, 5 vol% H_2_O).

In view of the importance of the C-H deprotonation mediated by the 2-hydroxypyridine ligand, which was highlighted by the computational and kinetic studies, we proceeded by varying the basicity of this ligand. Substituents on the heteroaromatic 2-hydroxypyridine ring enable a rational control of its basicity based on electronic effects. Electron-withdrawing substituents such as -CF_3_ and -NO_2_ on the C4 and C5 positions of the 2-hydroxypyridine were tested, which decrease the basicity of the ligand. In contrast, the -CH_3_ substituent increases the overall basicity of the ligand. The rate constants were obtained from the experimental time profiles for each substituted 2-hydroxypyridine ligand (Figure S12). When the logarithmic ratio of the rate constants was plotted in function of the Hammett parameters of the corresponding substituents (either in *para*- or *meta*-position relative to the 2-hydroxy functional group), a linear free-energy relationship was revealed between the catalytic activity and the basicity of the ligand (Figure 4A). Electron-donating groups with negative σ_x_ values boost the catalytic activity, showing that the higher basicity of the substituted 2-hydroxypyridine ligand indeed promotes C-H deprotonation. The catalytic activity however strongly decreases with less basic 2-hydroxypyridines, as indicated by the lower rate constants at positive σ_x_ values. Furthermore, the linearity of the Hammett plot demonstrates that the basicity of the ligand is a key feature in this type of C-H activation and that the electron density of the 2-hydroxypyridine ligand can be controlled to maximize the turnover rate.

While the previous reactions were performed with bromobenzene as the coupling partner, the oxidative addition can also have a strong influence on the turnover rate as exemplified by the previously reported phosphine-free systems. Our kinetic model highlighted the importance of accelerating both potentially rate-determining steps, especially in the presence of less reactive aryl bromides (*i.e.* with electron-donating substituents). To this end, the catalytic performance was evaluated for a series of functionalized bromoarenes, bearing substituents on the *para*-position (Figures S9 and S10). The reaction was successful over the whole range of electron-poor to electron-rich reactants. However, a strong decrease in reaction rate was observed for the less reactive aryl bromides (*i.e.* 4-bromoanisole and 4-bromotoluene). The logarithmic ratios of the observed rate constants were compared with their corresponding Hammett substituent values (Figure 4B). This revealed that, on the one hand, the catalytic activity stagnates for electron-poor bromoarenes, indicating that the oxidative addition is in that case not limiting the overall turnover rate. On the other hand, the reaction rate shows a decrease with stronger electron-donating substituents, which can be explained by a slower oxidative addition. An evaluation of different phosphine ligands however indicated that this negative effect can be mitigated with a more strongly electron-donating phosphine ligand. The conversion of electron-rich aryl bromides was increasingly problematic in the case of triphenyl phosphite as a weaker electron-donating ligand, whereas the use of tri-*tert*-butyl phosphine or the dialkylbiaryl ligand SPhos only led to a minor decrease in reaction rate for electron-donating substituents. Like in the traditional cross-coupling reactions, these ligands promote the oxidative addition step, but without strongly affecting the rate of C-H activation. The influence of varying bromoarene reactants could be satisfactorily incorporated in the kinetic model by modifying the rate constant of oxidative addition (k_1_) via the Hammett equation (see SI for derivation).

To conclude, we maximized the turnover rate of a Pd catalyst for C-H arylation, comprising a phosphine ligand for oxidative addition and a 2-hydroxypyridine to accelerate the C-H activation, via rational ligand modification. The underlying mechanism of a model C-H arylation of imidazo[1,2-*a*]pyridine with bromobenzene was first investigated via DFT calculations. Based on these insights, a kinetic model was devised that is in strong agreement with the experimentally obtained turnover rates in this reaction. Both ligands could be further modified in a predictable way to accelerate either the oxidative addition or C-H activation as the rate-determining steps, effectively balancing both steps to achieve the highest turnover. We believe that such model-driven ligand optimizations will provide inspiration for researchers in academia and industry to accelerate the turnover rates in homogeneous transition metal catalysis.

## Limitations of study

This study illustrates the kinetic and computational modeling of the direct arylation of imidazo[1,2-*a*]pyridines as an example for model-driven ligand optimization. The applicability of the acquired insights should be evaluated on a case-by-case basis and the conclusions drawn in this research should not be rigorously expanded to other catalytic systems.

## STAR★Methods

### Key resources table

**Table:** 

REAGENT or RESOURCE	SOURCE	IDENTIFIER
Bromobenzene, ≥99.5%	Sigma-Aldrich	Cat# 16350
Imidazo[1,2-*a*]pyridine, 99%	Sigma-Aldrich	Cat# 275778
Tris(dibenzylideneacetone)dipalladium(0), 97%	Sigma-Aldrich	Cat# 328774
Potassium hydroxide, anhydrous ≥99.95% trace metals basis	Sigma-Aldrich	Cat# 757551
*N*,*N*-dimethylacetamide, anhydrous 99.8%	Sigma-Aldrich	Cat# 271012
2-Hydroxypyridine, 97%	Sigma-Aldrich	Cat# H56800
Triphenylphosphine, 99%	Sigma-Aldrich	Cat# T84409
2-Hydroxy-5-methylpyridine, 97%	Sigma-Aldrich	Cat# 593427
2-Hydroxy-4-methylpyridine, 98+%	Sigma-Aldrich	Cat# 11322927
2-Hydroxy-5-(trifluoromethyl)pyridine, 97%	Sigma-Aldrich	Cat# 442801
4-Trifluoromethyl-2-hydroxypyridine, 97%	ThermoScientific	Cat# 15491559
5-Fluoro-2-hydroxypyridine, 97%	Sigma-Aldrich	Cat# 753181
4-Fluoro-2-hydroxypyridine, 97%	ThermoScientific	Cat# 15434317
2-Hydroxy-5-nitropyridine, 97%	Sigma-Aldrich	Cat# H48808
4-Bromobenzotrifluoride, 99%	Sigma-Aldrich	Cat# 152692
1-Bromo-4-nitrobenzene, 99%	Sigma-Aldrich	Cat# 167150
4-Bromotoluene, 98%	Sigma-Aldrich	Cat# B82200
4-Bromoanisole, ≥99.0%	Sigma-Aldrich	Cat# B56501
Tri(*t*-butyl)phosphine, 98%	Sigma-Aldrich	Cat# 570958
Triphenyl phosphite, 97%	Sigma-Aldrich	Cat# T84654
SPhos, 98% (2-Dicyclohexylphosphino-2′,6′-dimethoxybiphenyl)	Sigma-Aldrich	Cat# 637072

**Software and algorithms**		

Gaussian09	Frish et al.	https://gaussian.com
ChemDraw Ultra 12.0	PerkinElmer	https://www.perkinelmer.com/category/chemdraw

**Other**		

Shimadzu GC-2010 Pro with FID detection	Shimadzu	https://www.shimadzu.com/an/products/gas-chromatography/index.html

### Resource availability

#### Lead contact

Further information and requests should be directed to the lead contact, Dirk De Vos (dirk.devos@kuleuven.be).

#### Materials availability

This study did not generate new unique materials. The chemicals used in this study were obtained from standard commercial suppliers and used as received.

### Method details

#### Computational methods

Density functional theory (DFT) calculations were performed using the Gaussian 16 A03 program. The geometries of all stationary points including the intermediates (I) and transition states (TS) were optimized using the B3LYP functional with SDD pseudopotential as implemented in Gaussian. The calculations included the G3BJ dispersion correction. The effect of the solvent (DMA) was also taken into account with the SCRF method. Frequency calculations were performed to evaluate the optimized structures: a single negative frequency was obtained for all the transition states, and for the intermediates positive frequencies were found exclusively. The single point energy calculations were performed with the PWPB95-D3 functional with the Def2TZVP and Def2QZVPP basis sets for light and heavy atoms respectively. This method was found to be exceptionally suitable in the benchmark study for Pd-based systems performed by Grimme et al.^30^

#### Experimental procedure for the kinetic study

In a 10 mL glass vial, 0.11 mmol (6.17 mg) of finely grinded potassium hydroxide (KOH) pellets was dissolvent in 1 mL of aequeous *N*,*N*-dimethylacetamide solvent containing 5 vol % of H_2_O. Imidazo[1,2-*a*]pyridine (0.1 mmol, 10.1 μL) and bromobenzene (0.1 mmol, 10.5 μL) were added volumetrically by means of calibrated syringe. The reaction mixture was provided with a chemically resistant magnetic stirring bar. The glass vial was covered with a crimp cap equipped with a septum, through which argon flow was bubbled with a Schlenk line. Similarly, Pd_2_dba_3_ (0.01 mmol, 9.16 mg) with triphenylphosphine (0.02 mmol, 5.25 mg) and 2-hydroxypyridine (0.1 mmol, 9.51 mg) was weighed in a glass vial equipped with a septum and purged with argon gas. Thereafter, purged DMA solvent (5 mL) was added volumetrically with a syringe. After full dissolution of the solids in the catalyst stock solution, either via stirring or sonication, 50 μL of the solution containing Pd_2_dba_3_, triphenylphosphine and 2-hydroxypyridine was transferred to the reaction mixture under inert conditions via a calibrated syringe. The reaction vial was then heated to 120 °C in a heating block with magnetic stirring (500 rpm).

The reaction time was accurately timed and the reaction mixture was regularly sampled and transferred to GC vials. The samples were analyzed via gas chromatography (GC). The GC analysis was performed on a Shimadzu GC-2010 Pro equipped with a CP-Sil 5 CB column and flame ionization detector. Accurate quantitative analysis and product identification via GC was performed with the corresponding calibration curves, obtained with the pure reactant or product as a reference.

#### Synthetic procedure to prepare the deuterium labeled reactant

To determine the kinetic isotope effect of the reaction, imidazo[1,2-*a*]pyridine was labeled with deuterium isotope on its C3 position. The procedure for deuterium labeling was adapted from a recent literature report by Hartwig and co-workers^34^ for the C-H bond deuteration of five-membered aromatic heterocycles. Silver carbonate (0.3 mmol, 82.7 mg) and JohnPhos (0.6 mmol, 179.0 mg) were weighed in a glass vial equipped with a stirring bar, after which imidazo[1,2-*a*]pyridine (3 mmol, 300 μL) and CD_3_OD (6 mL) were added. The vial was closed with a septum and purged with argon gas. Next, the vial was heated to 60 °C under magnetic stirring for 48 h. During work-up, a solid metal scavenger was added to the product mixture (SiliaMetS DMT), followed by filtration through Celite. The solvent was evaporated in vacuo, yielding imidazo[1,2-*a*]pyridine-d_2_ in 90% yield (81% deuterated on C3 position). The NMR spectra were recorded on a Bruker Avance III HD 400 MHz spectrometer in DMSO-d_6_ as the solvent. The corresponding kinetic profiles for both the imidazo[1,2-*a*]pyridine with and without deuterium labeling were compared. The ratio of both observed kinetic parameters was determined, corresponding to a KIE of 7.6.

## Data Availability

• This study did not generate any large datasets. The cartesian coordinates of the intermediate Pd complexes and involved transition states are directly included the Supplemental Information under Data S1. The kinetic time profiles obtained in this study to determine the observed rate constants are also given in Supplemental Information as referenced in the main text. • This paper does not report original code. • Any additional information required to reanalyze the data reported in this paper is available from the lead contact upon request.
