# Supplementary material for: Rational ligand modification maximizes turnover rate in a model Pd-catalyzed C-H arylation
Source: iScience. 2022 Dec 10;26(1):105790. doi: 10.1016/j.isci.2022.105790 (PMC9803853; doi:10.1016/j.isci.2022.105790)
Supplement: Document S1. Figures S1–S14, Table S1 and Data S1 [file mmc1.pdf]

iScience, Volume *26*

Supplemental information

Rational ligand modification maximizes turnover rate in a model Pd-catalyzed C-H arylation

Igor Beckers and Dirk De Vos

Table of contents

1. Kinetic Experiments 2

2. Isotope Labelling and Kinetic Isotope Effect 7

3. Mathematical Derivation of Kinetic Models 10

4. Structure-Activity Relationships 13

5. Computational Details and Cartesian Coordinates 16

1. Experiments Related to Kinetic Study of the C-H Arylation Reaction

1.1 Standard reaction conditions

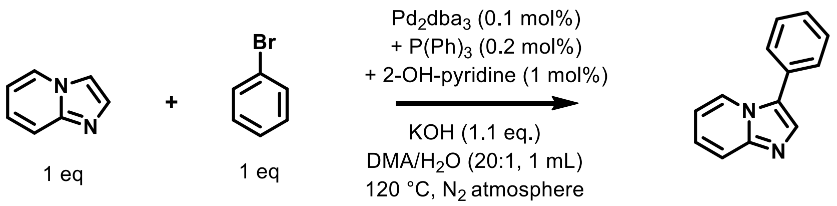

Table S1. Screening of the organic solvent in the coupling reaction of bromobenzene and imidazo[1,2-*a*]pyridine. Reaction conditions: Pd_2dba_3 (0.0001 mmol), PPh_3 (0.0002 mmol), 2-hydroxypyridine (0.001 mmol), imidazo[1,2-*a*]pyridine (0.1 mmol), bromobenzene (0.1 mmol), KOH (0.11 mmol) in aqueous DMA (1 mL, 5 vol% H_2O). Related to Figure 3C in the main text.

Entry	Time (h)	GC Yield (%)	[IP] (M)	ln([IP])
**1**	0	0	0.1000	-2.3026
**2**	0.5	30.8	0.0692	-2.6704
**3**	1	42.4	0.0576	-2.8538
**4**	2	57.8	0.0422	-3.1653
**5**	3	67.2	0.0328	-3.4173

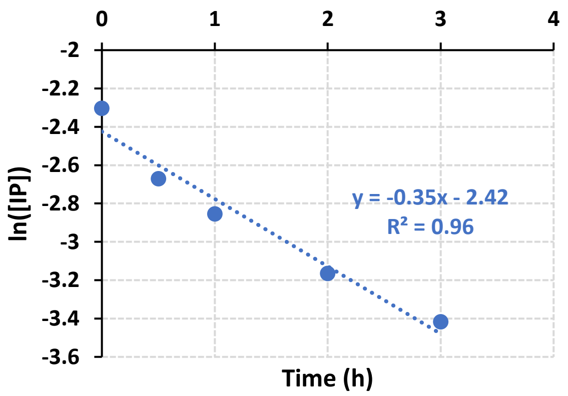

Figure S1. Linear regression of the natural logarithm of the imidazo[1,2-a]pyridine reactant concentration (M) in function of time (h) indicating a first-order kinetic profile. Reaction conditions: Pd_2dba_3 (0.0001 mmol), PPh_3 (0.0002 mmol), 2-hydroxypyridine (0.001 mmol), imidazo[1,2-*a*]pyridine (0.1 mmol), bromobenzene (0.1 mmol), KOH (0.11 mmol) in aqueous DMA (1 mL, 5 vol% H_2O). Related to Figure 3C in the main text.

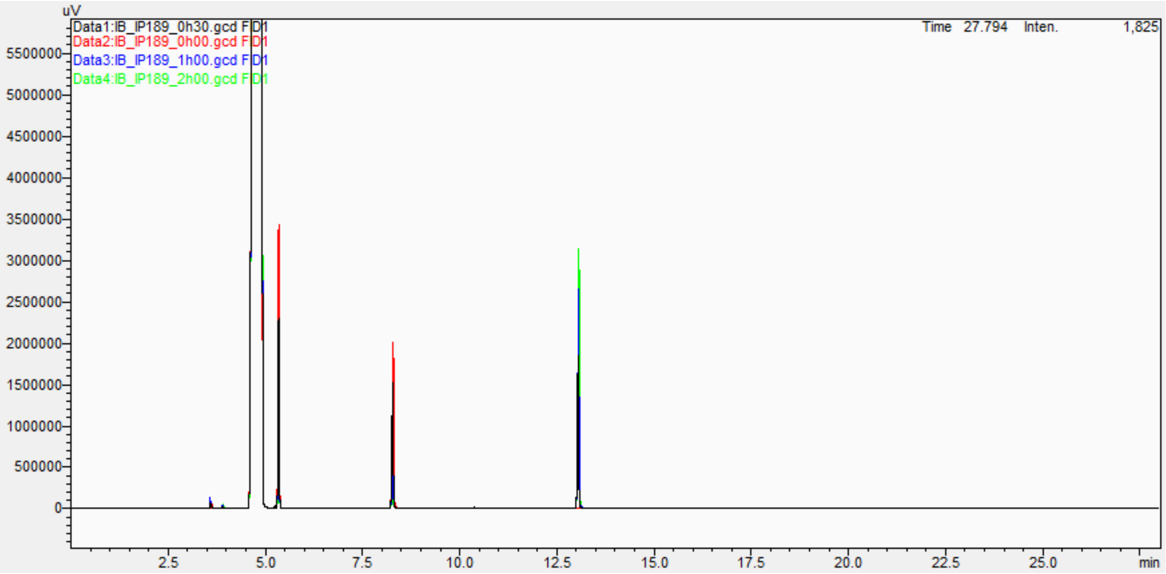

Figure S2. Full chromatograms obtained upon GC analysis of the crude reaction mixture after the reaction times of 0h (red), 30 min (black), 1h (blue) and 2h (green). Reaction conditions: Pd_2dba_3 (0.0001 mmol), PPh_3 (0.0002 mmol), 5-methyl-2-hydroxypyridine (0.001 mmol), imidazo[1,2-*a*]pyridine (0.1 mmol), bromobenzene (0.1 mmol), KOH (0.11 mmol) in aqueous DMA (1 mL, 5 vol% H_2O). Related to Figure 3 in the main text.

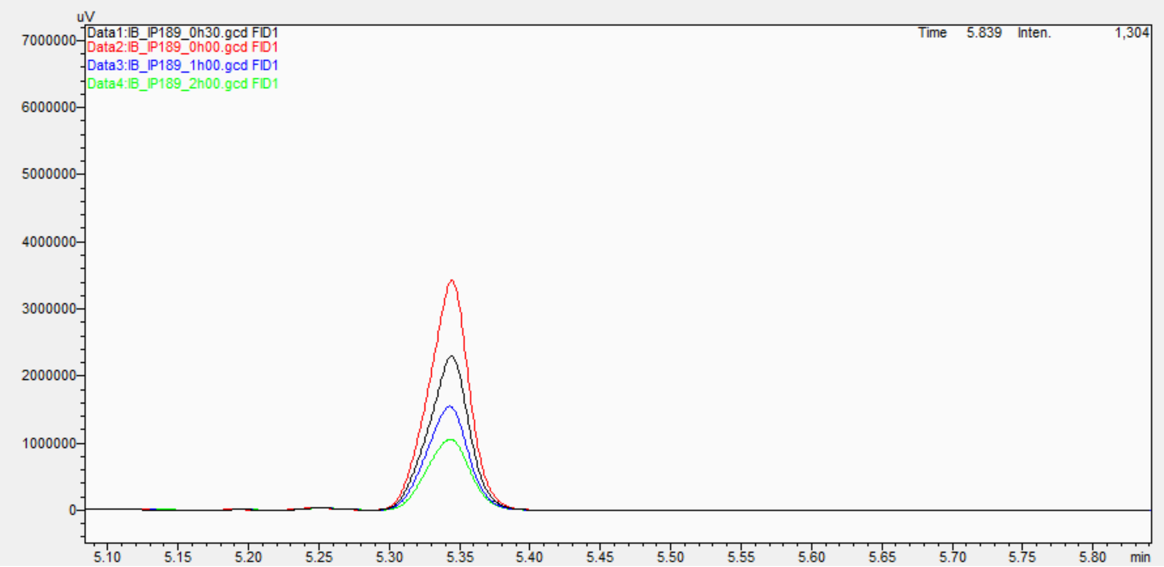

Figure S3. Chromatogram zoomed on the bromobenzene reactant peak, obtained upon GC analysis of the crude reaction mixture after the reaction times of 0h (red), 30 min (black), 1h (blue) and 2h (green). Reaction conditions: Pd_2dba_3 (0.0001 mmol), PPh_3 (0.0002 mmol), 5-methyl-2-hydroxypyridine (0.001 mmol), imidazo[1,2-*a*]pyridine (0.1 mmol), bromobenzene (0.1 mmol), KOH (0.11 mmol) in aqueous DMA (1 mL, 5 vol% H_2O). Related to Figure 3 in the main text.

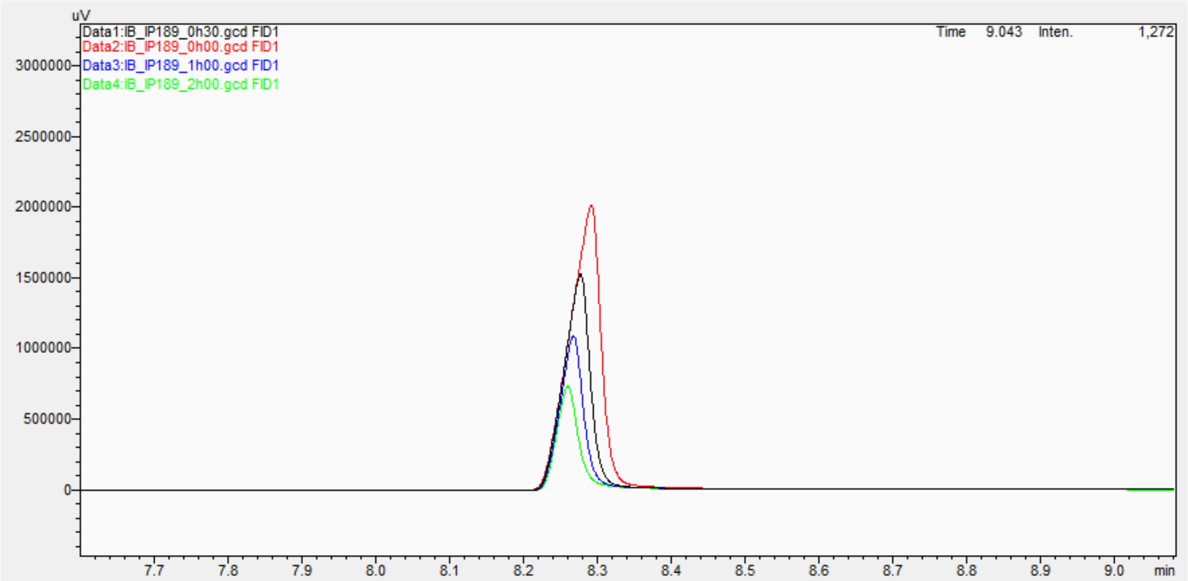

Figure S4. Chromatogram zoomed on the imidazo[1,2-*a*]pyridine reactant peak, obtained upon GC analysis of the crude reaction mixture after the reaction times of 0h (red), 30 min (black), 1h (blue) and 2h (green). Reaction conditions: Pd_2dba_3 (0.0001 mmol), PPh_3 (0.0002 mmol), 5-methyl-2-hydroxypyridine (0.001 mmol), imidazo[1,2-*a*]pyridine (0.1 mmol), bromobenzene (0.1 mmol), KOH (0.11 mmol) in aqueous DMA (1 mL, 5 vol% H_2O). Related to Figure 3 in the main text.

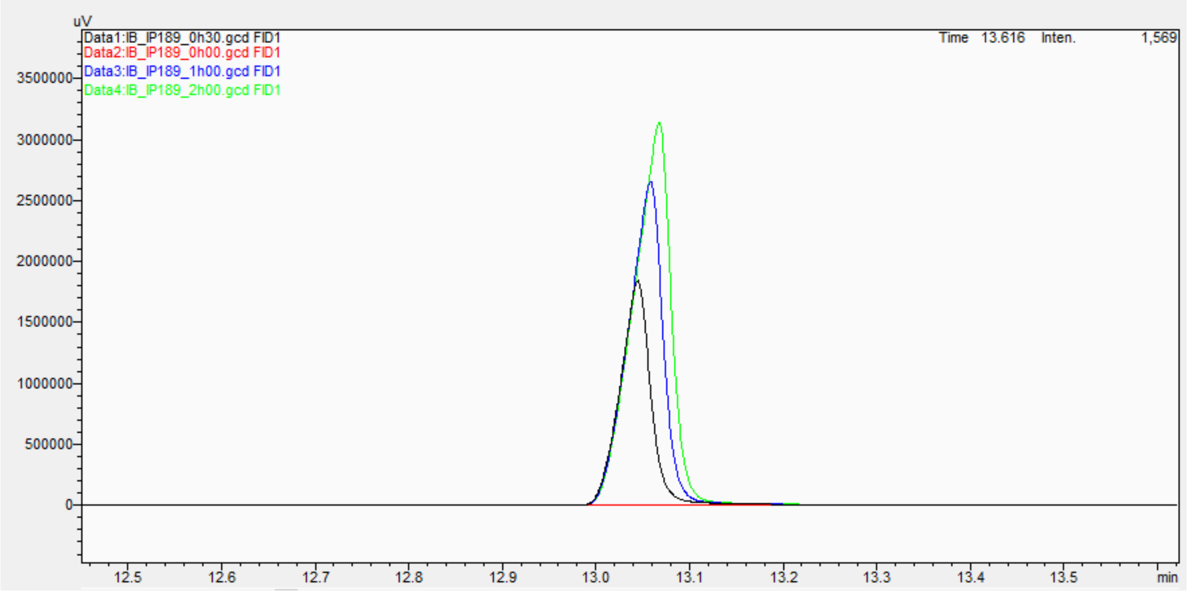

Figure S5. Chromatogram zoomed on the 3-phenyl-imidazo[1,2-*a*]pyridine product peak, obtained upon GC analysis of the crude reaction mixture after the reaction times of 0h (red), 30 min (black), 1h (blue) and 2h (green). Reaction conditions: Pd_2dba_3 (0.0001 mmol), PPh_3 (0.0002 mmol), 5-methyl-2-hydroxypyridine (0.001 mmol), imidazo[1,2-*a*]pyridine (0.1 mmol), bromobenzene (0.1 mmol), KOH (0.11 mmol) in aqueous DMA (1 mL, 5 vol% H_2O). Related to Figure 3 in the main text.

1.2 Catalyst concentration

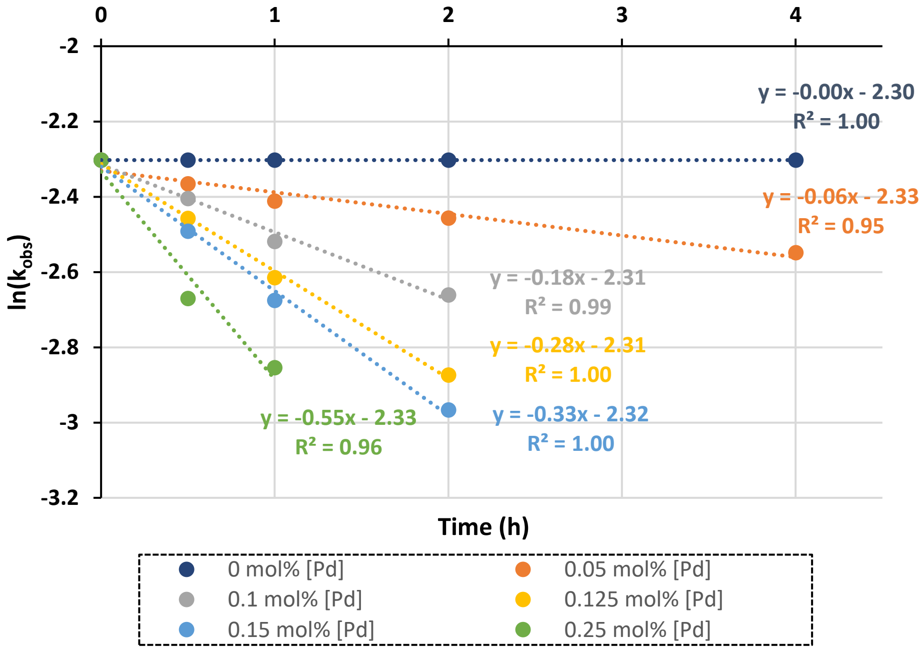

Figure S6. Linear regression of the natural logarithm of the imidazo[1,2-*a*]pyridine reactant concentration (M) in function of time (h) for different catalyst concentrations. Reaction conditions: Pd_2dba_3 (0-0.000125 mmol), PPh_3 (0-0.00025 mmol), 2-hydroxypyridine (0-0.00125 mmol), imidazo[1,2-*a*]pyridine (0.1 mmol), bromobenzene (0.1 mmol), KOH (0.11 mmol) in aqueous DMA (1 mL, 5 vol% H_2O). Related to Figure 3D in the main text.

1.3 Temperature

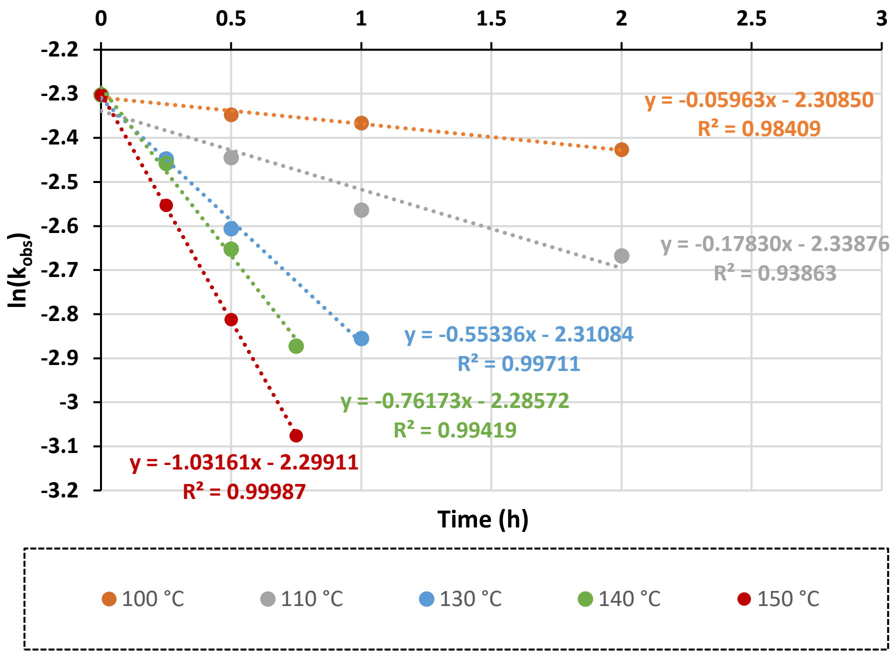

Figure S7. Logarithm of the imidazo[1,2-*a*]pyridine concentration in function of time for a range of reaction temperatures. Reaction conditions: Pd_2dba_3 (0.000125 mmol), PPh_3 (0.00025 mmol), 2-hydroxypyridine (0.00125 mmol), imidazo[1,2-*a*]pyridine (0.1 mmol), bromobenzene (0.1 mmol), KOH (0.11 mmol) in aqueous DMA (1 mL, 5 vol% H_2O). Related to Figure 3D in the main text.

2. Isotope Labelling and Kinetic Isotope Effect Related to Kinetic Study of the C-H Arylation Reaction

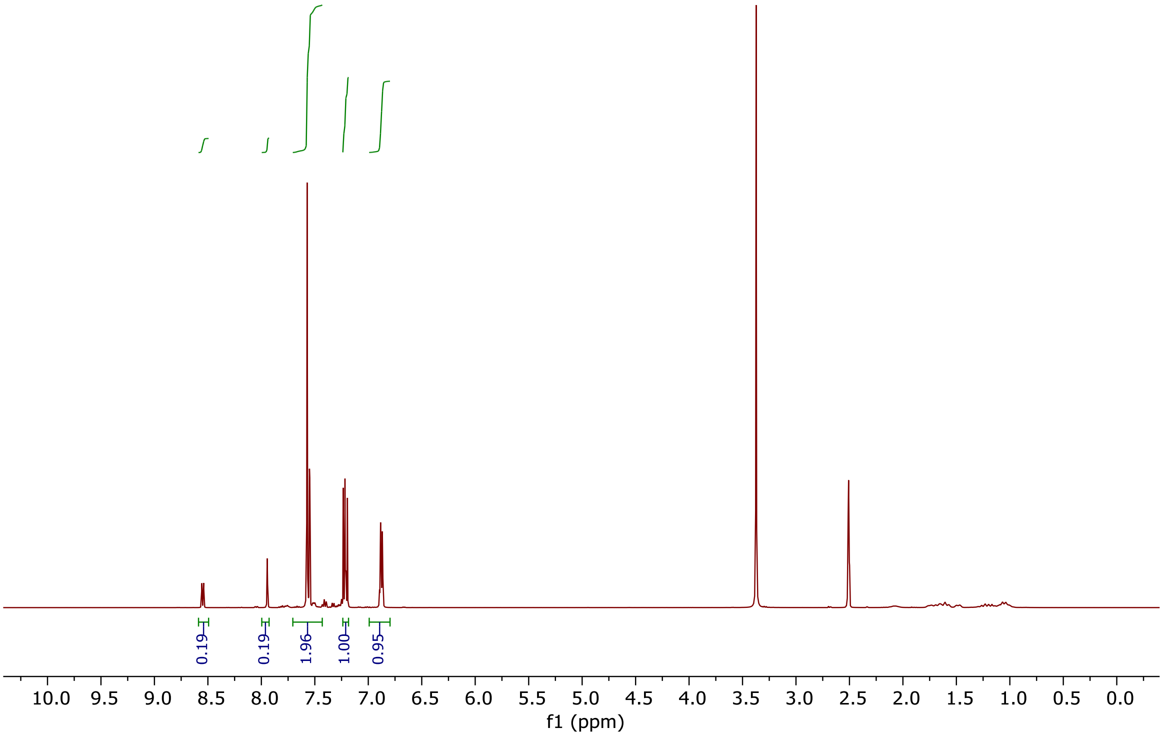

Figure S8. ^{1}H NMR spectrum of deuterium labelled imidazo[1,2-*a*]pyridine. Related to the kinetic isotope effect described in the main text.

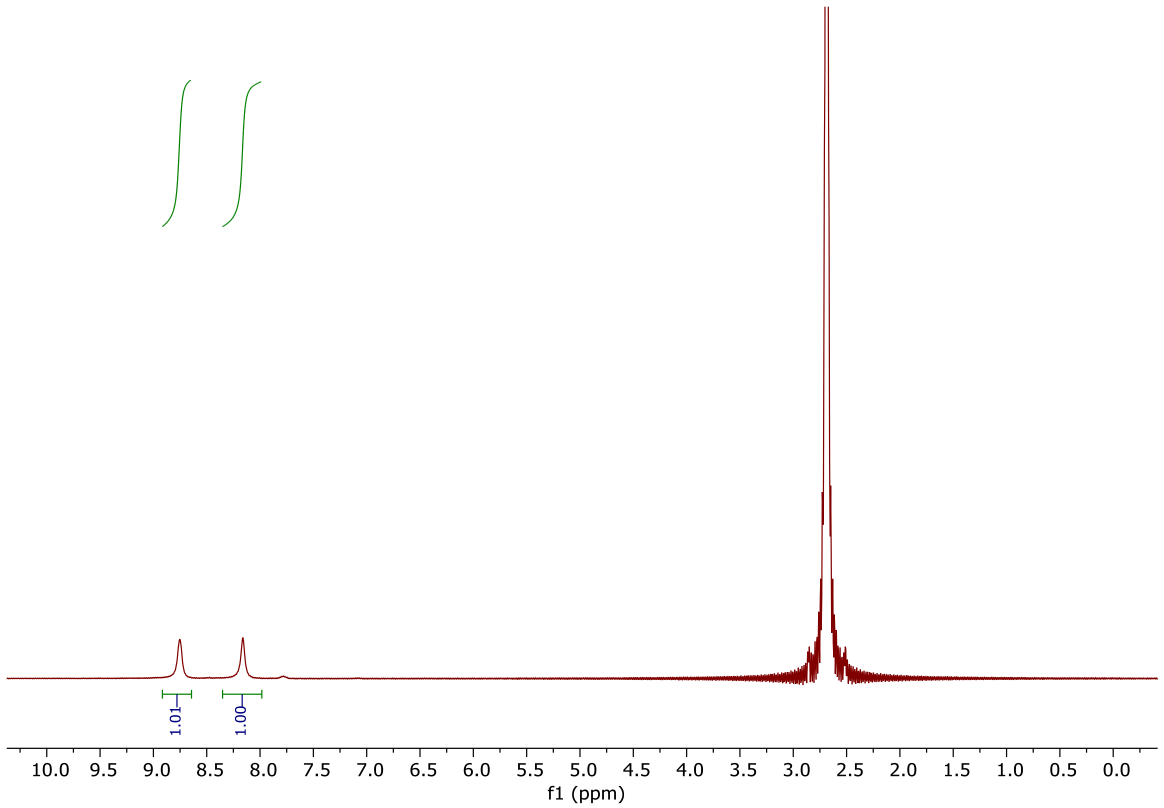

Figure S9. ^{2}H NMR spectrum of deuterium labelled imidazo[1,2-a]pyridine. Related to the kinetic isotope effect described in the main text (section Kinetic Study of the C-H Arylation Reaction).

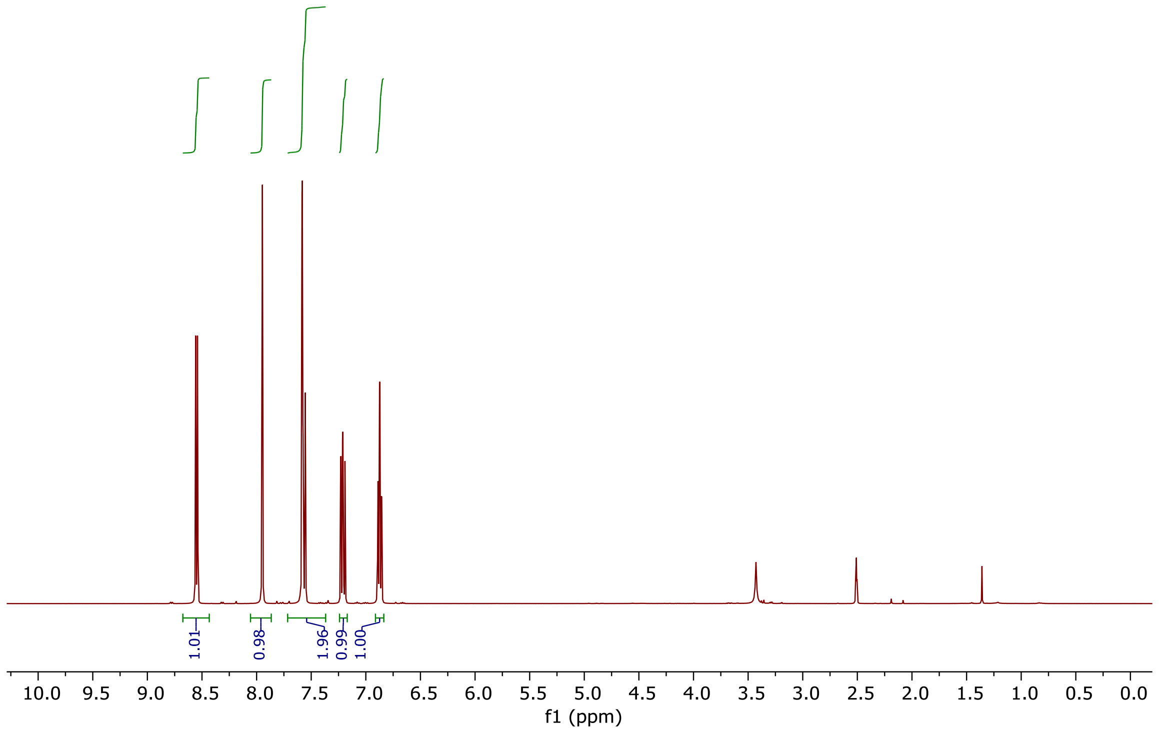

Figure S10. ^{1}H NMR spectrum of imidazo[1,2-a]pyridine without isotope labelling. Related to the kinetic isotope effect described in the main text (section Kinetic Study of the C-H Arylation Reaction).

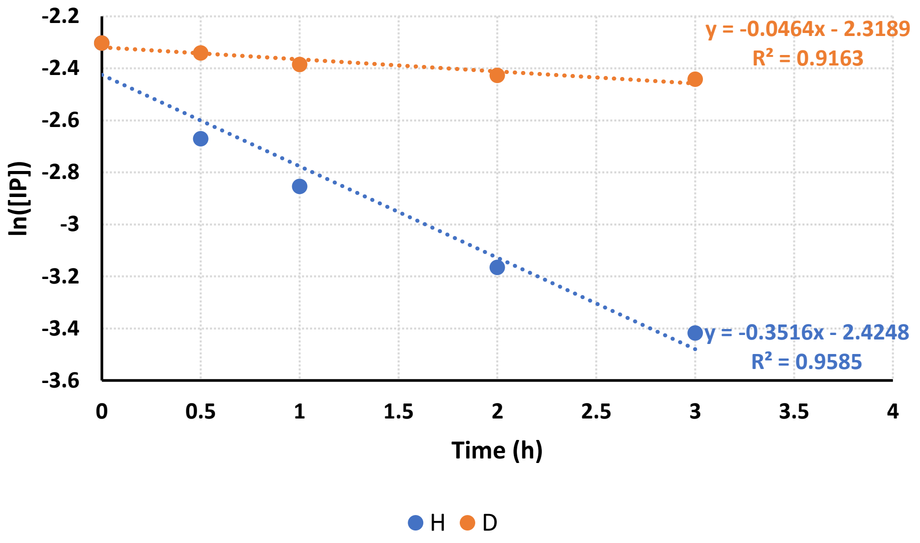

Figure S11. Observed kinetic profiles for the reaction with or without deuterium labelled imidazo[1,2-a]pyridine reactant. Reaction conditions: Pd_2dba_3 (0.000125 mmol), PPh_3 (0.00025 mmol), 2-hydroxypyridine (0.00125 mmol), imidazo[1,2-a]pyridine (0.1 mmol), bromobenzene (0.1 mmol), KOH (0.11 mmol) in aqueous DMA (1 mL, 5 vol% H2O). Related to the kinetic isotope effect described in the main text (section Kinetic Study of the C-H Arylation Reaction).

3. Mathematical Derivation of Kinetic Models Related to the Section Kinetic Study of the C-H Arylation Reaction

3.1 Kinetic model for standard reaction with bromobenzene (related to the mathematical model given in Figure 3B and 3C)

ArBr = Arylbromide

IP = imidazopyridine

P = product

Assumptions:

1) -d[ArBr]/dt = -d[IP]/dt = d[P]/dt
2) Reductive elimination and halide abstraction steps are very fast

1st order in both potentially slow steps; steady state in the concentration of the [Ar-Pd] intermediate:

$$-d[ArBr]/dt = k_1 [Pd^0] [ArBr]$$

$$d[P]/dt = k_2 [Ar\text{-}Pd] [IP]$$

so $$k_1 [Pd^0] [ArBr] = k_2 [Ar\text{-}Pd] [IP]$$

with [ArBr]=[IP] for equal initial concentrations;

and $$[Pd^0] + [Ar\text{-}Pd] = [Pd]_{TOT}$$

due to mass balance in Pd

so $$[Pd^0] = (k_2/k_1) [Ar\text{-}Pd]$$

so $$[ArPd] = k_1/(k_1 + k_2) [Pd]_{TOT}$$

and $$[Pd^0] = k_2/(k_1 + k_2) [Pd]_{TOT}$$

<u>Integration of the rate equation:</u>

$$-d[ArBr]/dt = k_1 [Pd^0] [ArBr] = \frac{k_1.k_2}{k_1+k_2} [Pd]_{TOT} [ArBr]$$

$$-d[IP]/dt = \frac{k_1.k_2}{k_1+k_2} [Pd]_{TOT} [IP]$$

$$[IP] = [IP]_0 \exp(- \frac{k_1.k_2}{k_1+k_2} [Pd]_{TOT}\ t)$$

So, with both potentially slow elementary steps that are 1st order in [ArBr] and [IP] respectively, the overall reaction also shows a (pseudo) first order in which the rate constant assumes the form $\frac{k_1.k_2}{k_1+k_2}$.

3.2 Kinetic model for standard reaction with aryl bromides with non-coordinating substituents (related to the mathematical model given in Figure 4C)

$k_{eff,\,X}$ = observed rate constant for aryl bromide with substituent X

$k_{1,\,X}$ = rate constant of oxidative addition for aryl bromide with substituent X

Assume that oxidative addition (k_1) follows the Hammett relationship:

$$\log(k_{1,X}/k_{1,H}) = \rho\,\sigma_x$$

$$k_{1,X} = k_{1,H}\,10^{\rho\sigma x}$$

with a positive ρ-value due to oxidative addition.

Inserting the Hammett relationship into the overall kinetic equation:

$$k_{eff,\,X} = \frac{k_{1,X}.k_2}{k_{1,X}+k_2} = \frac{k_{1,H}10^{\rho\sigma_x}.k_2}{k_{1,H}10^{\rho\sigma_x}+k_2}$$

Divide by $k_{eff,H}$ and take the logarithm:

$$\log\left(\frac{k_{eff,X}}{k_{eff,H}}\right) = \log\left(\frac{k_{1,X}.k_2}{k_{eff,H}.(k_{1,X}+k_2)}\right) = \log\left(\frac{k_{1,H}10^{\rho\sigma_x}.k_2}{k_{eff,H}.(k_{1,H}10^{\rho\sigma_x}+k_2)}\right)$$

$$= \rho\sigma_X + \log(k_{1,H}) + \log(k_2) - \log(k_{eff,H}) - \log(k_{1,H}10^{\rho\sigma_x} + k_2)$$

Because $\log(k_{eff,H}) = \log(k_{1,H}) + \log(k_2) - \log(k_{1,H} + k_2)$

The equation simplifies to

$$\log\left(\frac{k_{eff,X}}{k_{eff,H}}\right) = \rho\sigma_X + \log(k_{1,H} + k_2) - \log(k_{1,H}10^{\rho\sigma_x} + k_2)$$

On the one hand, when $\sigma_X > 0$ (*i.e.* for sufficiently electron-withdrawing substituents on the aryl bromide), $10^{\rho\sigma x}$ assumes large values. In that case, it holds that:

$$k_{1,H}10^{\rho\sigma_x} \gg k_2$$

So $\log(k_{1,H}10^{\rho\sigma_x} + k_2) \sim \log(k_{1,H}10^{\rho\sigma_x}) = \log(k_{1,H}) + \rho\sigma_x$

And the equation simplifies to:

$$\log\left(\frac{k_{eff,X}}{k_{eff,H}}\right) = \log(k_{1,H} + k_2) - \log(k_{1,H})$$

Thus, the Hammett plot stagnates to a constant value showing that the C-H activation step becomes rate-limiting: the overall rate becomes independent of the electron-withdrawing nature of the aryl bromide substituent, and is controlled by the rate of C-H activation (k_2).

On the other hand, when $\sigma_X < 0$ (*i.e.* for electron-donating substituents on the aryl bromide), the exponential $10^{\rho\sigma x}$ becomes small. It holds that:

$$k_{1,H}10^{\rho\sigma_x} \ll k_2$$

So $$\log\left(k_{1,H}10^{\rho\sigma_x} + k_2\right) \sim \log(k_2)$$

And the equation simplifies to:

$$\log\left(\frac{k_{eff,X}}{k_{eff,H}}\right) = \rho\sigma_X + \log\left(k_{1,H} + k_2\right) - \log\left(k_2\right)$$

Thus, for strongly electron-donating substituents on the aryl bromide reactant, the effective reaction rate is dependent on the electron-donating nature of the substituents on the aryl halide. Eventually, the rate of oxidative addition becomes rate-limiting and may assume a linear Hammett relationship .

4. Supplemental Data Related to the Section Structure-Activity Relationships between Ligand and Catalytic Activity

4.1. 2-Hydroxypyridine Ligand

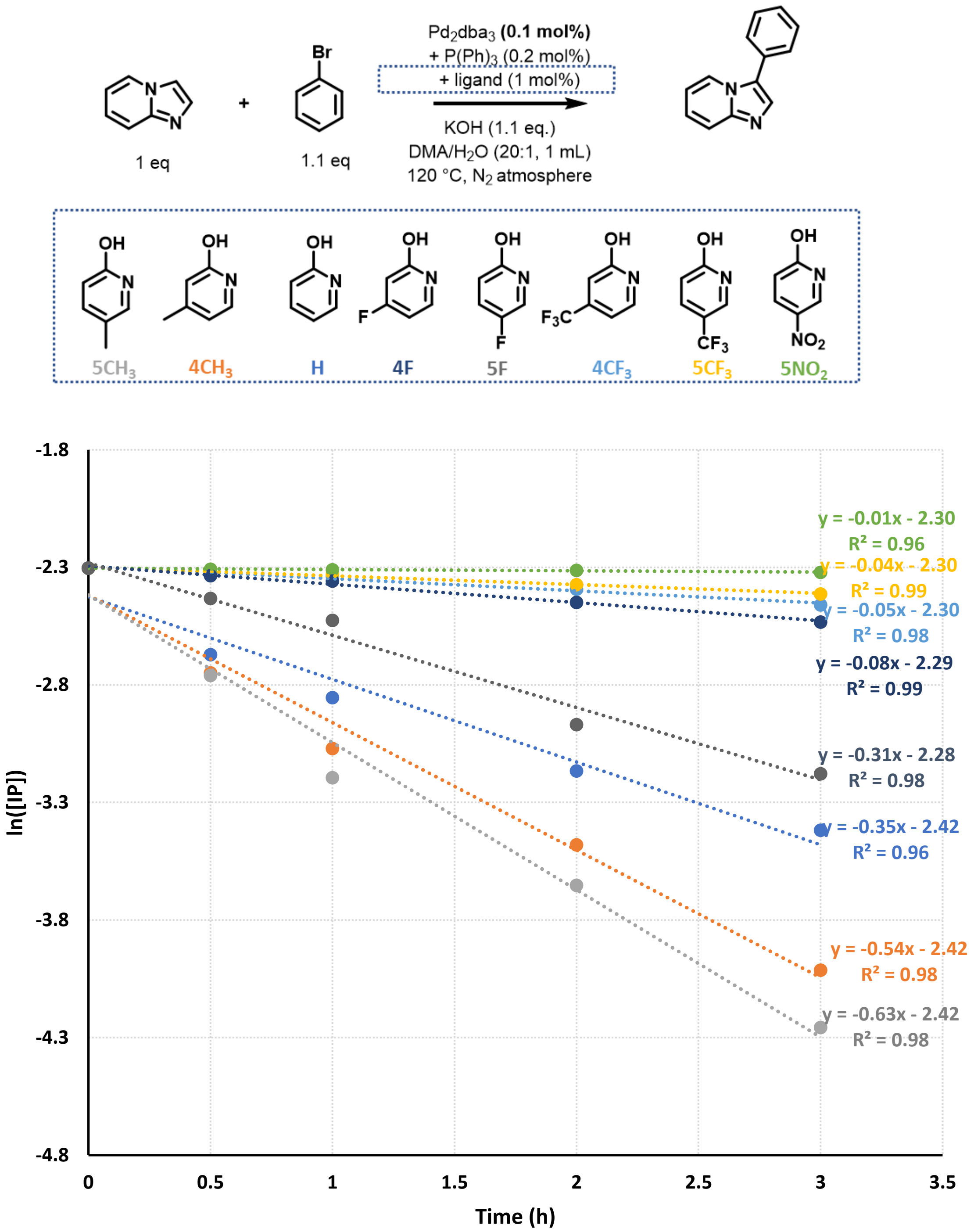

Figure S12. Logarithm of the imidazo[1,2-a]pyridine concentration in function of time for various substituted 2-hydroxypyridines. Reaction conditions: Pd_2dba_3 (0.000125 mmol), PPh_3 (0.00025 mmol), substituted 2-hydroxypyridine (0.00125 mmol), imidazo[1,2-a]pyridine (0.1 mmol), bromobenzene (0.1 mmol), KOH (0.11 mmol) in aqueous DMA (1 mL, 5 vol% H_2O). Related to Figure 4B in the main text

4.2. Aryl Bromide Reactants

4.2.1. Triphenylphosphine ligand

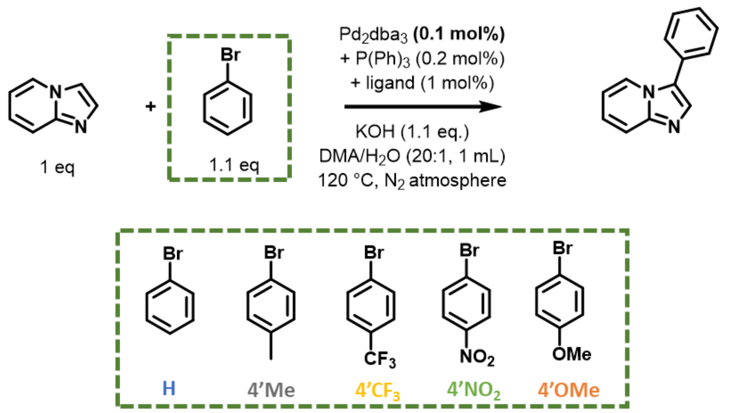

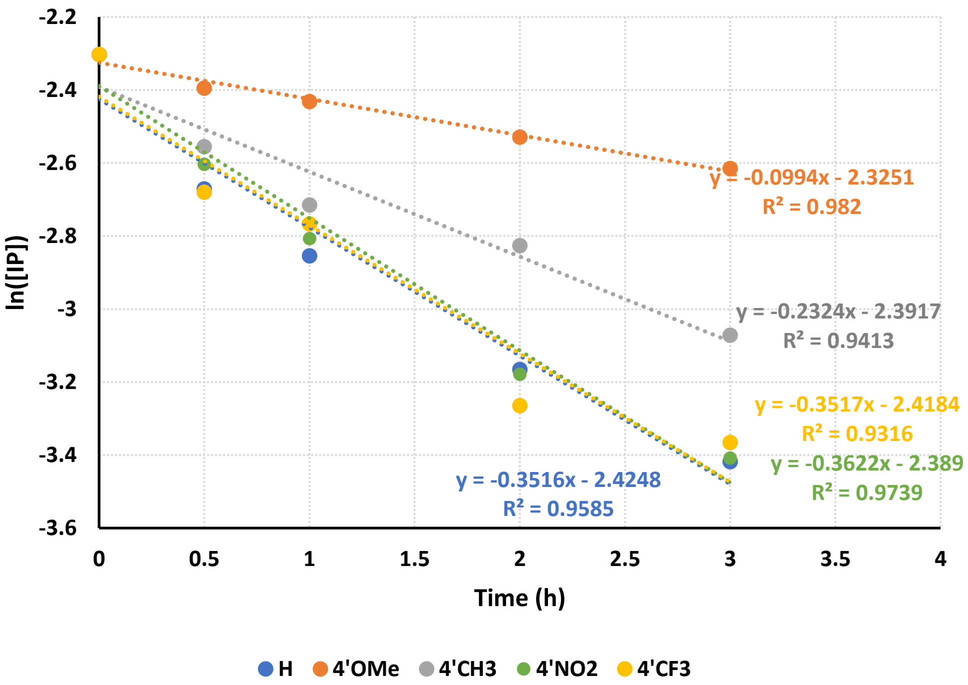

Figure S13. Logarithm of the imidazo[1,2-a]pyridine concentration in function of time for various arylbromide reactants. Reaction conditions: Pd_2dba_3 (0.000125 mmol), PPh_3 (0.00025 mmol), 2-hydroxypyridine (0.00125 mmol), imidazo[1,2-a]pyridine (0.1 mmol), substituted bromobenzene (0.1 mmol), KOH (0.11 mmol) in aqueous DMA (1 mL, 5 vol% H_2O). Related to Figure 4C in the main text

4.2.2. Other phosphine ligands

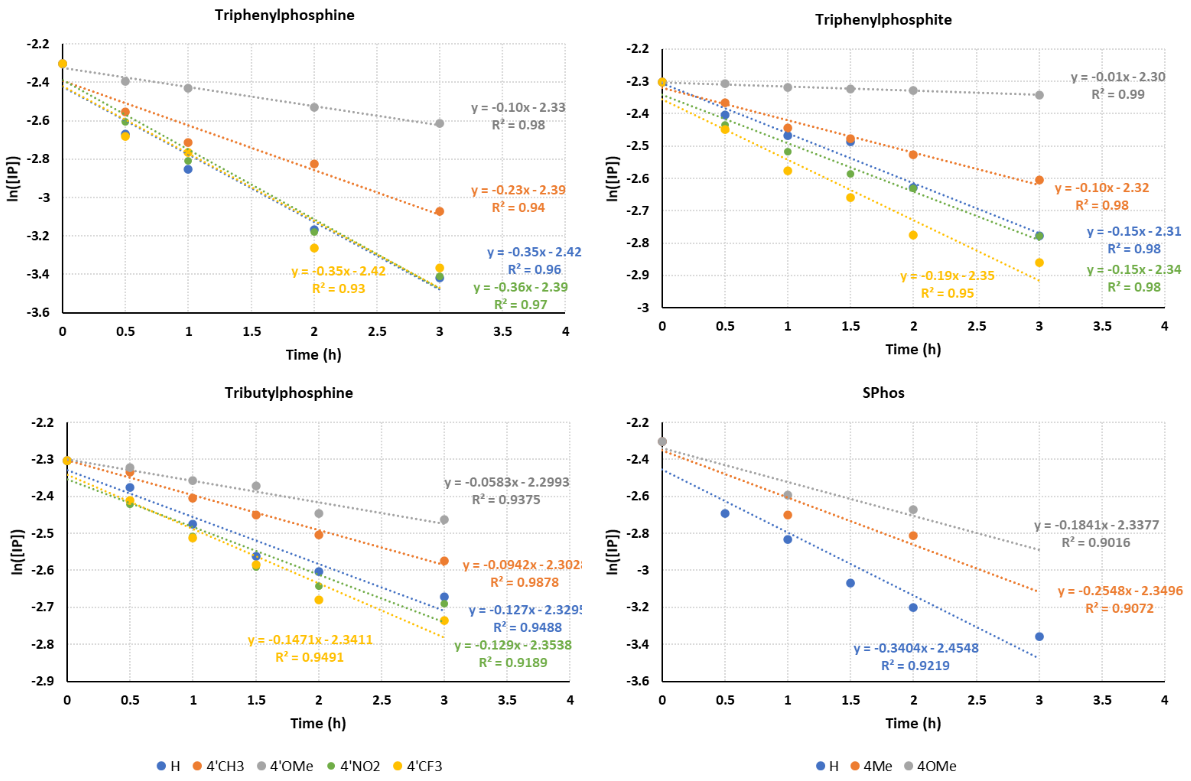

Figure S14. Logarithm of the imidazo[1,2-a]pyridine concentration in function of time for various arylbromide reactants, with a) triphenylphosphine, b) triphenylphosphite, c) tributylphosphine and d) SPhos as the phosphine ligand. Reaction conditions: Pd_2dba_3 (0.000125 mmol), phosphine ligand (0.00025 mmol), 2-hydroxypyridine (0.00125 mmol), imidazo[1,2-a]pyridine (0.1 mmol), substituted bromobenzene (0.1 mmol), KOH (0.11 mmol) in aqueous DMA (1 mL, 5 vol% H_2O). Related to Figure 4C in the main text

Data S1. Cartesian Coordinates Related to the Section Computational Modelling and Catalytic Cycle (Figures 1 and 2 in the main text)

I_0

H	0.05890	3.85451	2.34475
C	-0.34386	4.26339	1.42599
N	-0.05831	3.56959	0.28360
C	-0.53731	4.03221	-0.90197
C	-1.31650	5.19794	-1.00417
H	-1.67519	5.52031	-1.97412
C	-1.60615	5.90154	0.17267
H	-2.20847	6.80335	0.12907
C	-1.11332	5.42962	1.41110
H	-1.32209	5.95176	2.33735
O	-0.23623	3.29722	-2.02189
H	0.30208	2.48775	-1.72429
Pd	0.94186	1.72217	0.23251
P	1.83368	-0.39290	0.03488
H	0.86280	-3.20670	0.61588
C	0.41385	-2.88101	-0.31716
C	-0.46847	-3.73982	-1.00239
H	-0.69349	-4.71970	-0.59044
C	-1.05729	-3.33008	-2.21372
H	-1.74158	-3.99107	-2.73831
C	-0.76350	-2.05446	-2.73781
H	-1.21965	-1.72849	-3.66833
C	0.10593	-1.19354	-2.04691
H	0.31050	-0.19999	-2.43802
C	0.70126	-1.60361	-0.83505
H	6.88964	-0.93415	-2.69954
C	5.93283	-0.84172	-2.19264
C	5.00476	-1.90123	-2.23799
H	5.24445	-2.81297	-2.77837
C	3.76513	-1.78333	-1.58117
H	3.05214	-2.60181	-1.62276
C	3.44638	-0.60196	-0.88232
C	4.37133	0.46196	-0.84680
H	4.11149	1.37973	-0.32355
C	5.61390	0.34123	-1.49531
H	6.32285	1.16409	-1.46401
C	2.14729	-1.23086	1.67657
C	1.27404	-0.92234	2.74199
H	0.48025	-0.19404	2.59045
C	1.43214	-1.54721	3.99181
H	0.75411	-1.30690	4.80619
C	2.47399	-2.47667	4.18832
H	2.60170	-2.95594	5.15525
C	3.35300	-2.77741	3.12932
H	4.16022	-3.48976	3.27665
C	3.18727	-2.15952	1.87454
H	3.86915	-2.39820	1.06415
C	-4.10440	-1.04527	0.02102
C	-2.72837	-0.78562	0.08259
C	-2.30297	0.55447	0.15435
C	-3.24108	1.60322	0.16462
C	-4.61845	1.31570	0.10213
C	-5.06422	-0.02076	0.02860
H	-6.12411	-0.24697	-0.02153
H	-2.90266	2.63419	0.21964
H	-5.34912	2.11955	0.10884
H	-2.00439	-1.59154	0.07030
H	-1.23468	0.77644	0.19426
Br	-4.70916	-2.92096	-0.08912

TS_1

Pd	0.98277	0.07915	-0.10240
Br	1.96922	-1.98750	-1.98269
P	-1.42786	0.33764	-0.02917
C	1.58612	-3.32137	2.65748
C	0.46896	-3.43400	1.80038
C	0.45851	-2.80010	0.54368
C	1.56500	-1.99192	0.17130
C	2.72890	-1.93884	0.98593
C	2.71181	-2.57899	2.24152
H	1.58868	-3.82435	3.62087
H	-0.39364	-4.02437	2.10042
H	-0.38414	-2.91819	-0.12853
H	3.61365	-1.41210	0.64699
H	3.58840	-2.50786	2.88127
C	-3.94080	-3.00026	-2.21363
C	-4.26857	-2.65135	-0.88933
C	-3.54166	-1.64587	-0.21841
C	-2.47575	-0.98658	-0.86441
C	-2.14209	-1.34966	-2.19006
C	-2.87441	-2.34381	-2.86373
H	-4.50319	-3.77353	-2.73086
H	-5.08616	-3.15401	-0.37865
H	-3.81117	-1.38617	0.80108
H	-1.31052	-0.86343	-2.69524
H	-2.60994	-2.60943	-3.88415
C	-3.17665	0.61099	4.34207
C	-3.86726	1.23221	3.28185
C	-3.36409	1.15668	1.96809
C	-2.16214	0.46520	1.70298
C	-1.47104	-0.15076	2.76995
C	-1.97723	-0.08188	4.08225
H	-3.56499	0.67073	5.35571
H	-4.79087	1.77229	3.47417
H	-3.90530	1.64630	1.16359
H	-0.54053	-0.67871	2.57812
H	-1.43566	-0.56007	4.89448
C	-2.86543	4.35142	-2.05078
C	-3.69657	3.21505	-2.10971
C	-3.28771	2.00553	-1.51282
C	-2.03928	1.91921	-0.85991
C	-1.20490	3.05832	-0.81418
C	-1.61789	4.27024	-1.39876
H	-3.18235	5.28423	-2.51037
H	-4.65847	3.26808	-2.61353
H	-3.93881	1.13752	-1.56762
H	-0.23333	2.99747	-0.32862
H	-0.96738	5.14004	-1.35374
N	2.88308	1.26347	-0.12794
C	3.76718	1.32133	-1.16862
C	4.91490	2.12006	-1.13001
C	5.16646	2.89073	0.02901
C	4.26600	2.84040	1.10198
C	3.13478	2.01117	0.97606
O	2.22615	1.94149	2.00803
H	1.46770	1.34139	1.73165
H	4.41418	3.41350	2.00986
H	3.52995	0.70528	-2.02808
H	5.59066	2.13776	-1.97771
H	6.04954	3.51954	0.09151

I_1

H	2.26995	-0.04274	2.07379
C	2.94928	-0.10303	1.23259
N	2.42087	-0.57419	0.06790

C	3.21430	-0.66010	-1.01915
C	4.57547	-0.29879	-0.99451
H	5.18225	-0.38431	-1.88984
C	5.11259	0.18246	0.20558
H	6.15630	0.47545	0.25229
C	4.28438	0.28799	1.34386
H	4.66275	0.66254	2.28739
O	2.57747	-1.09802	-2.16192
H	3.18155	-1.17472	-2.93001
Pd	0.36436	-1.19914	-0.00769
P	-0.47472	0.99866	-0.01424
H	-2.38157	3.09351	0.93877
C	-2.06568	2.51806	1.80383
C	-2.54820	2.85545	3.08125
H	-3.23192	3.69183	3.19530
C	-2.14251	2.11356	4.20894
H	-2.51601	2.37526	5.19512
C	-1.25707	1.02889	4.05389
H	-0.94650	0.44841	4.91793
C	-0.78015	0.68794	2.77609
H	-0.11375	-0.16202	2.65453
C	-1.18074	1.43206	1.64936
H	3.80111	4.76154	-0.93098
C	2.98348	4.06738	-0.75737
C	2.16062	4.21354	0.37552
H	2.33934	5.02135	1.07967
C	1.10427	3.31113	0.60602
H	0.48172	3.42988	1.48658
C	0.87032	2.25730	-0.29695
C	1.69683	2.10990	-1.43093
H	1.53700	1.28379	-2.11802
C	2.74743	3.01249	-1.66198
H	3.38237	2.88651	-2.53441
C	-1.82551	1.45707	-1.21544
C	-3.13159	0.97210	-0.98859
H	-3.34587	0.35483	-0.12300
C	-4.16429	1.28690	-1.88660
H	-5.16634	0.90999	-1.70211
C	-3.90133	2.07674	-3.02392
H	-4.70106	2.31633	-3.71950
C	-2.59755	2.55622	-3.25245
H	-2.38513	3.17037	-4.12315
C	-1.56146	2.24864	-2.34987
H	-0.56720	2.64203	-2.53095
C	-1.54462	-1.79984	-0.10715
C	-2.07951	-2.13508	-1.37292
C	-3.41312	-2.56912	-1.50233
C	-4.24094	-2.66834	-0.36529
C	-3.71528	-2.34427	0.90153
C	-2.37719	-1.91551	1.02836
H	-1.99583	-1.66584	2.01441
H	-5.27381	-2.99302	-0.46455
H	-4.34145	-2.41995	1.78816
H	-1.46153	-2.04731	-2.26424
H	-3.80596	-2.81695	-2.48622
Br	1.11430	-3.65495	0.31636

I_1^-

H	2.37808	-0.15824	2.06467
C	3.02217	-0.18369	1.19002
N	2.43051	-0.56963	0.02741
C	3.15344	-0.61886	-1.16535
C	4.55787	-0.28768	-1.10028
H	5.12865	-0.33980	-2.02299
C	5.14815	0.10072	0.09758

H	6.20501	0.35719	0.12474
C	4.36785	0.16686	1.28448
H	4.79245	0.47265	2.23416
O	2.54858	-0.92753	-2.27453
Pd	0.40870	-1.18991	-0.03805
P	-0.44455	0.99240	0.00947
H	-2.50568	2.94338	0.99240
C	-2.15632	2.36996	1.84579
C	-2.66855	2.64413	3.12718
H	-3.40810	3.42964	3.25491
C	-2.21998	1.90611	4.24084
H	-2.61572	2.11931	5.23012
C	-1.26193	0.88760	4.06718
H	-0.91718	0.30990	4.92021
C	-0.75689	0.60903	2.78513
H	-0.03411	-0.19069	2.64505
C	-1.19988	1.34993	1.67195
H	3.57974	5.06030	-0.74469
C	2.81560	4.30193	-0.59644
C	1.91186	4.40953	0.47820
H	1.97441	5.25110	1.16272
C	0.92412	3.42503	0.67366
H	0.23780	3.51601	1.50896
C	0.83978	2.32774	-0.20468
C	1.74873	2.21625	-1.27707
H	1.70959	1.35539	-1.93802
C	2.73070	3.20192	-1.47335
H	3.43136	3.10390	-2.29786
C	-1.78345	1.45116	-1.20820
C	-3.09173	0.95853	-1.01314
H	-3.32127	0.33548	-0.15567
C	-4.10584	1.26921	-1.93376
H	-5.10971	0.88614	-1.77252
C	-3.82184	2.06120	-3.06447
H	-4.60694	2.29709	-3.77790
C	-2.51490	2.54540	-3.26414
H	-2.28496	3.15842	-4.13131
C	-1.49846	2.24256	-2.33796
H	-0.50023	2.63585	-2.49811
C	-1.50477	-1.80985	-0.15097
C	-2.03445	-2.14188	-1.42177
C	-3.36333	-2.58601	-1.56611
C	-4.19990	-2.70082	-0.43661
C	-3.68483	-2.38264	0.83636
C	-2.35084	-1.94460	0.97480
H	-1.97990	-1.69931	1.96647
H	-5.22961	-3.03299	-0.54610
H	-4.31690	-2.47051	1.71811
H	-1.41165	-2.04233	-2.30923
H	-3.74677	-2.82933	-2.55518
Br	1.17151	-3.65553	0.29104

TS_2

H	2.64113	1.54504	1.09001
C	3.22031	1.09384	0.29082
N	2.58277	0.18111	-0.47830
C	3.22514	-0.46185	-1.52610
C	4.58520	-0.12452	-1.80920
H	5.08855	-0.62014	-2.63340
C	5.23423	0.82498	-1.01511
H	6.26976	1.08346	-1.22392
C	4.55302	1.45038	0.06236
H	5.03952	2.18724	0.69146
O	2.49481	-1.35474	-2.16884
Pd	0.70623	-0.80194	-0.53617

P	-0.75953	0.86273	0.14620
H	-3.04345	1.98274	1.72915
C	-2.48932	1.30896	2.37620
C	-2.90177	1.12115	3.70755
H	-3.76851	1.65679	4.08458
C	-2.19087	0.24161	4.54974
H	-2.51136	0.09662	5.57795
C	-1.07026	-0.45322	4.05392
H	-0.52433	-1.13914	4.69585
C	-0.65822	-0.27004	2.72139
H	0.19244	-0.82399	2.33110
C	-1.36612	0.61336	1.88378
H	1.99082	5.93051	0.02828
C	1.46309	4.98119	0.05935
C	0.73583	4.61475	1.20810
H	0.69987	5.27937	2.06680
C	0.05190	3.38409	1.25102
H	-0.50306	3.10999	2.14232
C	0.10293	2.51365	0.14630
C	0.84312	2.87530	-0.99923
H	0.91890	2.18560	-1.83490
C	1.51369	4.10794	-1.04628
H	2.08223	4.37859	-1.93139
C	-2.31697	1.15698	-0.83946
C	-3.40189	0.26779	-0.68526
H	-3.33467	-0.56242	0.00918
C	-4.57309	0.44709	-1.43856
H	-5.40233	-0.24325	-1.31119
C	-4.66845	1.50837	-2.36149
H	-5.57390	1.64419	-2.94685
C	-3.58417	2.39226	-2.51963
H	-3.64846	3.21588	-3.22545
C	-2.41098	2.21801	-1.76031
H	-1.59052	2.91665	-1.88405
C	-0.92779	-1.90402	-0.88332
C	-1.39739	-1.99911	-2.21430
C	-2.50155	-2.81394	-2.53527
C	-3.16696	-3.53580	-1.52362
C	-2.70879	-3.44466	-0.19318
C	-1.60007	-2.63349	0.12399
H	-1.25626	-2.57661	1.15216
H	-4.02628	-4.15615	-1.76704
H	-3.21289	-3.99902	0.59604
H	-0.91215	-1.42721	-3.00344
H	-2.84687	-2.87374	-3.56543
Br	1.95822	-2.91882	1.36561

I_2

H	3.39184	1.29259	0.12256
C	3.78580	0.28373	0.06934
N	2.88276	-0.71737	-0.05021
C	3.28588	-2.03880	-0.11837
C	4.66723	-2.36462	-0.08571
H	4.97433	-3.40317	-0.14596
C	5.59570	-1.32230	0.03128
H	6.65793	-1.55095	0.06062
C	5.16157	0.02518	0.11465
H	5.86808	0.84174	0.20983
O	2.26202	-2.89676	-0.20725
Pd	0.77231	-1.21757	-0.12076
P	-0.46942	0.72867	0.01017
H	-2.64199	2.41425	1.20080
C	-2.09419	1.95080	2.01580
C	-2.46352	2.21775	3.34658
H	-3.29058	2.89156	3.55118

C	-1.76174	1.61495	4.40970
H	-2.04930	1.82153	5.43690
C	-0.69095	0.74012	4.13864
H	-0.15057	0.26844	4.95436
C	-0.32362	0.46882	2.80906
H	0.49455	-0.21449	2.59648
C	-1.02340	1.07495	1.74645
H	2.81517	5.15530	-1.67346
C	2.19390	4.32629	-1.34613
C	1.37482	4.46458	-0.20837
H	1.36237	5.39959	0.34449
C	0.56838	3.39239	0.21845
H	-0.05753	3.50766	1.09752
C	0.58806	2.17586	-0.48978
C	1.41528	2.03318	-1.62334
H	1.45143	1.08500	-2.15270
C	2.21209	3.10769	-2.05405
H	2.84761	2.99121	-2.92710
C	-2.00694	0.89511	-1.02737
C	-3.17846	0.21073	-0.63977
H	-3.18973	-0.39127	0.26258
C	-4.33691	0.29953	-1.42813
H	-5.23509	-0.22908	-1.12161
C	-4.33158	1.06066	-2.61476
H	-5.22786	1.12531	-3.22567
C	-3.16018	1.73736	-3.00429
H	-3.14738	2.32845	-3.91575
C	-1.99905	1.65627	-2.21174
H	-1.10668	2.19275	-2.51720
C	-0.97331	-2.19113	-0.14556
C	-1.52003	-2.63410	-1.37213
C	-2.70900	-3.38990	-1.39724
C	-3.37543	-3.70617	-0.19563
C	-2.83646	-3.27059	1.03163
C	-1.64458	-2.51662	1.05631
H	-1.25289	-2.17557	2.01162
H	-4.29864	-4.28001	-0.21573
H	-3.34222	-3.50854	1.96500
H	-1.03151	-2.37906	-2.31035
H	-3.11783	-3.71960	-2.34996

TS_3

Pd	0.51754	-0.59274	-0.06316
N	-0.44747	-2.53451	0.02052
O	1.13865	-3.23290	-1.54087
C	0.02396	-3.49858	-0.84132
C	-0.65458	-4.73909	-0.97510
P	-1.51994	0.63035	-0.00079
C	1.51831	1.14892	-0.10278
C	4.56779	-1.12362	0.73888
N	3.97732	-1.79041	1.77947
C	2.69785	-2.07104	1.34909
C	2.41517	-1.59147	0.04145
N	3.65418	-0.99081	-0.32893
H	2.00599	-2.60472	1.98652
C	3.99081	-0.35113	-1.49911
H	1.77238	-2.41127	-0.91578
C	5.87416	-0.59441	0.59995
C	6.21518	0.04993	-0.58466
C	5.26126	0.16983	-1.64543
H	6.57399	-0.70066	1.42104
H	7.20941	0.46678	-0.71092
H	5.52241	0.67605	-2.56744
H	3.21976	-0.28274	-2.25398
C	-1.78547	-4.99038	-0.19604

C	-2.23043	-4.01315	0.72714
C	-1.53471	-2.80799	0.80017
H	-1.84387	-2.02518	1.48063
H	-2.31229	-5.93534	-0.29228
H	-3.09393	-4.18034	1.36028
H	-0.26694	-5.46683	-1.67885
C	2.23138	1.58101	1.03976
C	2.98234	2.77339	1.01967
H	2.22269	0.97950	1.94669
C	3.04224	3.55577	-0.15181
H	3.52386	3.08379	1.91127
C	2.33960	3.13327	-1.29792
H	3.62409	4.47425	-0.17026
C	1.58265	1.94557	-1.26801
H	2.37163	3.72861	-2.20834
H	1.03384	1.64990	-2.15931
C	-2.86938	0.06645	-1.15046
C	-2.18273	0.46215	1.73934
C	-1.46704	2.47214	-0.28720
C	-4.09257	0.76765	-1.22943
C	-5.10035	0.33102	-2.10576
C	-4.89075	-0.80542	-2.91386
C	-3.66498	-1.49450	-2.84836
C	-2.65660	-1.05891	-1.96964
H	-1.70975	-1.58412	-1.93275
H	-5.67028	-1.14174	-3.59194
H	-3.49099	-2.36276	-3.47756
H	-4.25200	1.66113	-0.63278
H	-6.03906	0.87486	-2.16069
C	-1.14032	3.34945	0.76391
C	-1.02356	4.72924	0.51903
H	-0.97780	2.97149	1.76783

C	-1.22780	5.23898	-0.77833
C	-1.55005	4.35946	-1.83038
C	-1.66465	2.97954	-1.58679
H	-1.70699	4.74310	-2.83477
H	-1.90595	2.30954	-2.40675
H	-0.77215	5.39996	1.33602
H	-1.13687	6.30535	-0.96649
C	-1.23999	0.51840	2.79019
C	-1.66004	0.39590	4.12539
C	-3.02425	0.20162	4.42063
C	-3.96289	0.13111	3.37302
C	-3.54467	0.26430	2.03504
H	-0.18470	0.64114	2.56470
H	-0.92781	0.44202	4.92657
H	-3.34978	0.09809	5.45201
H	-5.01456	-0.03003	3.59274
H	-4.27846	0.19182	1.24022

I_3

Pd	0.68196	-0.27648	0.01705
N	0.17248	-2.38275	0.04677
O	1.40983	-2.59830	-1.92782
C	0.64119	-3.17652	-0.94732
C	0.34929	-4.55001	-1.02337
P	-1.65420	0.35349	-0.04160
C	1.19680	1.66646	0.07617
C	4.93569	-0.88866	0.43270
N	4.48104	-1.84103	1.28259
C	3.08889	-1.78085	1.18814
C	2.63140	-0.79671	0.29457
N	3.83300	-0.22776	-0.19082
H	2.47228	-2.45090	1.76991
C	4.03463	0.77429	-1.12482

H	1.69939	-1.69091	-1.63312
C	6.25779	-0.49324	0.09299
C	6.44888	0.51921	-0.83669
C	5.31748	1.15592	-1.45247
H	7.08946	-0.99936	0.57173
H	7.45278	0.83220	-1.10694
H	5.46020	1.94398	-2.18357
H	3.14841	1.22062	-1.54981
C	-0.44175	-5.11318	-0.01487
C	-0.90887	-4.29896	1.04196
C	-0.58000	-2.94288	1.03832
H	-0.91324	-2.27491	1.82091
H	-0.68575	-6.17023	-0.04432
H	-1.51349	-4.70520	1.84404
H	0.74258	-5.13337	-1.84697
C	1.74077	2.22538	1.25761
C	2.07804	3.59269	1.33203
H	1.91776	1.59191	2.12399
C	1.89644	4.43127	0.21385
H	2.48965	3.99650	2.25512
C	1.36549	3.88736	-0.97342
H	2.16289	5.48424	0.26614
C	1.01281	2.52543	-1.03423
H	1.21339	4.52215	-1.84416
H	0.58437	2.13664	-1.95517
C	-2.76380	-0.52231	-1.24954
C	-2.29280	-0.05776	1.66691
C	-2.11457	2.14478	-0.29421
C	-4.14508	-0.23610	-1.31052
C	-4.96276	-0.89973	-2.24060
C	-4.40454	-1.84861	-3.12226
C	-3.02399	-2.11957	-3.07648
C	-2.20609	-1.45617	-2.14337
H	-1.14053	-1.65014	-2.12981
H	-5.03745	-2.36063	-3.84184
H	-2.58399	-2.83690	-3.76343
H	-4.57785	0.51369	-0.65457
H	-6.02498	-0.67591	-2.28063
C	-2.09050	3.05898	0.77559
C	-2.36478	4.41952	0.54741
H	-1.86581	2.72558	1.78304
C	-2.65663	4.87650	-0.75251
C	-2.67326	3.96267	-1.82445
C	-2.39984	2.60240	-1.59673
H	-2.89514	4.30537	-2.83147
H	-2.41186	1.90744	-2.43131
H	-2.34684	5.11714	1.38028
H	-2.86740	5.92804	-0.92763
C	-1.47958	0.32127	2.75978
C	-1.86767	0.00994	4.07366
C	-3.06428	-0.69714	4.30776
C	-3.86730	-1.08900	3.21949
C	-3.48470	-0.76848	1.90253
H	-0.53990	0.83720	2.58504
H	-1.23556	0.30679	4.90584
H	-3.36096	-0.94698	5.32263
H	-4.78463	-1.64532	3.39094
H	-4.10573	-1.09458	1.07595

TS$_4$

Pd	-0.78097	0.00155	0.20079
N	-0.41903	2.27224	0.30005
O	-0.57708	2.24133	-2.02784
C	-0.32176	2.94167	-0.86836
C	0.02556	4.30196	-0.94383

P	1.64105	-0.29262	-0.09520	H	-2.28942	-4.19565	-2.31474
C	-1.74727	-1.85339	0.15477	H	-2.35346	-1.77137	-1.94239
C	-4.94177	0.33173	0.76625	C	2.55143	1.19799	-0.74899
N	-4.60786	0.03453	2.05031	C	2.40167	-0.53494	1.59856
C	-3.29028	-0.38627	2.00587	C	2.33733	-1.72012	-1.07723
C	-2.74547	-0.37297	0.69783	C	2.62743	1.41284	-2.14101
N	-3.82168	0.10778	-0.08819	C	3.19898	2.59311	-2.64650
H	-2.78986	-0.73082	2.89887	C	3.68611	3.57785	-1.76360
C	-3.88422	0.38222	-1.44329	C	3.59809	3.37142	-0.37364
H	-0.70098	1.27823	-1.81087	C	3.03277	2.18720	0.13141
C	-6.15135	0.80612	0.20228	H	2.96869	2.03912	1.20521
C	-6.21818	1.04821	-1.16532	H	4.12271	4.49325	-2.15383
C	-5.06400	0.83803	-1.99203	H	3.96025	4.12962	0.31539
H	-6.99839	0.97571	0.85819	H	2.23953	0.66717	-2.83018
H	-7.13797	1.41184	-1.61211	H	3.25707	2.74564	-3.72086
H	-5.10167	1.05071	-3.05443	C	3.63094	-1.70612	-1.63886
H	-2.97459	0.25456	-2.01207	C	4.11204	-2.83328	-2.32945
C	0.27963	4.97726	0.25641	H	4.25673	-0.82261	-1.55380
C	0.18056	4.28575	1.48545	C	3.30593	-3.98307	-2.45796
C	-0.17114	2.93323	1.46380	C	2.01385	-3.99722	-1.89691
H	-0.25772	2.34831	2.37243	C	1.52970	-2.86833	-1.21098
H	0.55831	6.02609	0.23799	H	1.38250	-4.87635	-1.99411
H	0.37476	4.78197	2.42923	H	0.53254	-2.88214	-0.78878
H	0.09965	4.78715	-1.90938	H	5.10882	-2.81537	-2.76188
C	-1.39190	-2.76847	1.19719	H	3.67962	-4.85313	-2.99139
C	-1.34442	-4.14855	0.97214	C	1.56312	-0.47114	2.72893
H	-1.14468	-2.38460	2.18372	C	2.09692	-0.64450	4.01966
C	-1.66707	-4.68618	-0.29736	C	3.47419	-0.88570	4.18462
H	-1.04718	-4.81070	1.78240	C	4.31570	-0.95151	3.05412
C	-2.04046	-3.80591	-1.33010	C	3.78067	-0.77672	1.76553
H	-1.62775	-5.75823	-0.46937	H	0.49958	-0.28666	2.59669
C	-2.08015	-2.41529	-1.11367	H	1.44320	-0.59339	4.88617

H	3.88898	-1.02174	5.17981	H	4.43770	-0.82597	0.90225
H	5.37936	-1.13671	3.17692				
